# Analysis of the *Coptis chinensis* genome reveals the diversification of protoberberine-type alkaloids

**DOI:** 10.1038/s41467-021-23611-0

**Published:** 2021-06-02

**Authors:** Yifei Liu, Bo Wang, Shaohua Shu, Zheng Li, Chi Song, Di Liu, Yan Niu, Jinxin Liu, Jingjing Zhang, Heping Liu, Zhigang Hu, Bisheng Huang, Xiuyu Liu, Wei Liu, Liping Jiang, Mohammad Murtaza Alami, Yuxin Zhou, Yutao Ma, Xiangxiang He, Yicheng Yang, Tianyuan Zhang, Hui Hu, Michael S. Barker, Shilin Chen, Xuekui Wang, Jing Nie

**Affiliations:** 1 College of Pharmacy, Hubei University of Chinese Medicine, Wuhan, China; 2 Hubei Institute for Drug Control, Wuhan, China; 3 College of Plant Science and Technology, Huazhong Agricultural University, Wuhan, China; 4 Department of Ecology and Evolutionary Biology, University of Arizona, Tucson, AZ USA; 5 Wuhan Benagen Tech Solutions Company Limited, Wuhan, China; 6 National Resource Center for Chinese Materia Medica, China Academy of Chinese Medical Sciences, Beijing, China; 7 Jing Brand Chizhengtang Pharmaceutical Company Limited, Huangshi, China; 8 Institute of Chinese Materia Medica, China Academy of Chinese Medical Sciences, Beijing, China

**Keywords:** Genome evolution, Next-generation sequencing, Plant evolution, Secondary metabolism

## Abstract

Chinese goldthread (*Coptis chinensis* Franch.), a member of the Ranunculales, represents an important early-diverging eudicot lineage with diverse medicinal applications. Here, we present a high-quality chromosome-scale genome assembly and annotation of *C. chinensis*. Phylogenetic and comparative genomic analyses reveal the phylogenetic placement of this species and identify a single round of ancient whole-genome duplication (WGD) shared by the Ranunculaceae. We characterize genes involved in the biosynthesis of protoberberine-type alkaloids in *C. chinensis*. In particular, local genomic tandem duplications contribute to member amplification of a Ranunculales clade-specific gene family of the cytochrome P450 (CYP) 719. The functional versatility of a key CYP719 gene that encodes the (*S*)-canadine synthase enzyme involved in the berberine biosynthesis pathway may play critical roles in the diversification of other berberine-related alkaloids in *C. chinensis*. Our study provides insights into the genomic landscape of early-diverging eudicots and provides a valuable model genome for genetic and applied studies of Ranunculales.

## Abstract (web summary)

*Coptis chinensis* represents an early-diverging eudicot lineage with diverse medicinal applications. Here, the authors report its chromosome-scale genome assembly, infer a single ancient whole-genome duplication, and characterize the function of CYP719 in diversification of protoberberine-type alkaloids.

## Introduction

Species of the genus *Coptis* are among the most precious medicinal herbs benefiting human health worldwide^1^. The rhizomes of *Coptis* (Coptidis rhizome, CR), also known as “Huanglian” in Chinese, have been included in many classic formulae in traditional Chinese medicine (TCM) for thousands of years^1^. In the Han dynasty of China (202 B.C.–220 A.D.), the medicinal use of CR was first listed in Divine Farmer’s Materia Medica (Shennong Bencao Jing). During the Ming dynasty (1368 A.D.–1644 A.D.), the pharmacological effects and usage of Chinese goldthreads were already well documented in Compendium of Materia Medica (Bencao Gangmu) the Chinese encyclopedia of botany and medicine^1–3^. The active ingredients and agents of the bitter taste of CR are mainly protoberberine-type alkaloids such as berberine, coptisine, jatrorrhizine, palmatine, columbamine, epiberberine, and magnoflorine^4,5^. Various pharmacological properties of these alkaloids have been reported, including properties for treating generally infective and inflammatory diseases^3,6,7^, as well as properties related to the prevention and treatment of health problems related to cardiovascular, diabetes, cancer and the nervous system^8,9^.

The genus *Coptis*, together with other members of Ranunculaceae, are also important for their critical phylogenetic position in the tree of the extant flowering plants. More than two thousand species have been described in the Ranunculaceae and most of them are herbs. Of which, approximately 15 *Coptis* species are recognized, and most of them are restricted to smaller geographical regions of warm temperate climates and cold coniferous forests from eastern Asia to North America^10^. In China, *Coptis chinensis* (“Weilian” in Chinese) is a relatively widely distributed *Coptis* species, and its dried rhizomes are the most frequently used materials for “Huanglian” in TCM. The Ranunculaceae represents a key evolutionary lineage within the early evolving eudicots, and morphological or evolutionary transition species between core eudicots and other early diverging angiosperms or monocots^11,12^. However, the genomic resources of Ranunculaceae are limited^13,14^, hindering an in-depth understanding of the genomic landscape and the phylogenetic relationship of this important lineage with other eudicots.

The Japanese goldthread *C. japonica*, together with *Papaver somniferum* (opium poppy) and *Eschscholzia californica* (California poppy), have historically served as models for studying the biosynthesis of benzylisoquinoline alkaloids (BIAs)^15,16^. BIAs are a complex and diverse group of natural products, but their presence is thought to be restricted to certain plant families, including the Magnoliaceae, Ranunculaceae, Papaveraceae, and Berberidaceae^10,11^. In opium poppy, the most abundant alkaloids are morphinans (i.e., morphine, codeine, and thebaine), phthalideisoquinoline noscapine and the 1-benzylisoquinoline papaverine, which mainly occur in the latex of aerial tissues^15^. Genomic analysis of opium poppy has allowed the characterization of BIA gene clusters associated with the biosynthesis of noscapine and morphinan, suggesting potential selection pressure favouring quick formation and evolution of these alkaloids^16^. Comparatively, the evolutionary mechanisms underlying the occurrence of abundant protoberberine-type alkaloids in *Coptis* belowground rhizomes are still unclear, but this information is critical for our understanding of BIA diversification in plants.

Here, we assemble a high-quality chromosome-scale genome of *C. chinensis*. We perform a phylogenetic and comparative genomic analysis to resolve the phylogenetic position of *C. chinensis* as a key lineage within the early-evolving eudicots. We infer that one-round ancient whole-genome duplication (WGD) occur within *C. chinensis* evolution, and characterize genes from the cytochrome P450 (CYP) family which greatly contribute to the biosynthesis and diversification of protoberberine-type alkaloids in *Coptis*. The *C. chinensis* reference genome provides insights into the evolution of eudicots and is valuable for future genetic studies and medicinal applications of Ranunculales.

## Results

### Genome sequencing, assembly, and annotation

**Fig. 1:**
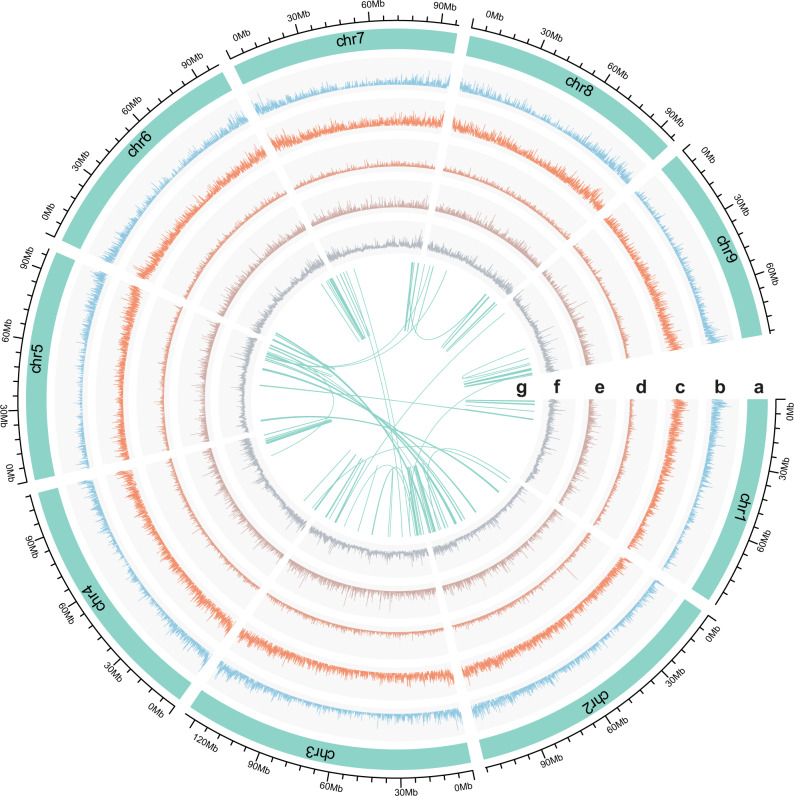
Landscape of genome assembly and annotation of *Coptis chinensis*. **a** Chromosome-scale pseudomolecules (chr1–chr7). **b** Density of transcripts. **c** Gene density. **d** Density of long retrotransposon terminal repeats. **e** Density of DNA transposable elements. **f** Density of biallelic heterozygous single-nucleotide polymorphisms. **g** Intragenomic synteny information.

*Coptis chinensis* is a diploid (2*n* = 2*x* = 18; Supplementary Fig. 1), with an estimated genome size of approximately 1.15 Gb/1C (Supplementary Fig. 2) according to flow cytometry and 1.02 Gb according to *k*-mer (*k* = 19) analysis (Supplementary Fig. 3). We performed Nanopore genome sequencing, which yielded approximately 6.6 million single-molecule long reads (average read length, 13.7 kb; Supplementary Fig. 4), with a total data volume of 85.9 Gb. After data filtering using the Oxford Nanopore Metrichor base caller, 73.8 Gb were retained for subsequent assembly. We used a hybrid assembly strategy in which the Nanopore long reads were corrected and assembled prior to using 108.9 Gb of Illumina short reads for further polishing (Supplementary Fig. 5 and Supplementary Table 1). The assembly resulted in 1801 sequence contigs (N50 = 806.6 kb), with a total size of 936.6 Mb (Supplementary Table 2). The assembly was further scaffolded with 436.7 million high-throughput chromatin conformation capture (Hi–C) paired-end reads. This yielded a final reference scaffold assembly of nine unambiguous chromosome-scale pseudomolecules covering approximately 97.9% (916.5 Mb) of the assembled genome size. The minimal length of the chromosome was greater than 85 Mb (Fig. 1a, Supplementary Fig. 6 and Supplementary Table 3).

To evaluate the quality of the assembled *C. chinensis* genome, we aligned the Illumina short reads to the assembled genome, resulting in a mapping rate of 98.6%. All the assembled transcripts from our Illumina RNA-seq reads generated from four different tissues were then added to the assembly (Fig. 1b, Supplementary Fig. 1 and Supplementary Table 4), in which 57,533 of 60,616 long transcripts (>1000 bp) were mapped, with >50% sequence coverage. Finally, we performed benchmarking universal single-copy orthologs (BUSCO) analysis based on plant gene models, which showed that 91.5% of the BUSCO sequences were completely present in the *C. chinensis* genome (including 90.2% of BUSCO genes presented in the annotation), while 6.9% and 1.6% were partially present or missing, respectively (Supplementary Table 5). Taken together, these results suggested a highly accurate and nearly complete *C. chinensis* genome assembly that was comparable to that of the recently reported opium poppy^16^ and stout camphor tree^17^ genomes.

We used an integrated strategy including evidence-based methods and ab initio gene prediction to annotate the protein-coding gene content of the *C. chinensis* genome. After removing nonfunctional annotations, a final set of 41,004 protein-coding genes was retained, of which 39,496 genes (~96.3%) could be assigned to nine pseudomolecules. We used tissue-specific RNA-seq data to confirm the expression of the genes. The average coding sequence length of the predicted genes was 969 bp, with an average of 4.6 exons per gene (Supplementary Table 6). The average gene density in *C. chinensis* was one gene per 22.8 kb, and the genes were unevenly distributed, being more abundant towards the chromosomal ends (Fig. 1c). Approximately, 87% of the genes were functionally annotated, of which 79.6% had significant hits in the InterPro database (Supplementary Table 7). We further annotated noncoding RNA genes (Supplementary Table 8), yielding 1134 transfer RNA (tRNA) genes, 492 ribosomal RNA (rRNA) genes, 1429 small nuclear RNA genes, and 106 microRNA genes.

### Repetitive content, genome heterozygosity, and demography

A total of 585 Mb (62.5%) of the assembly was masked and annotated as repetitive elements (Supplementary Table 9), of which 41.0% were long terminal repeat (LTR) retrotransposons (Fig. 1d), 5.3% were DNA transposable elements (Fig. 1e) and 2.7% were long interspersed nuclear elements (LINE). Interestingly, most LTRs were Gypsy elements (constituting 35.5% of the *C. chinensis* genome), and only 5.4% of the genome comprised Copia repeats. This was similar to the makeup of the *Liriodendron chinense* genome^18^ and the sunflower genome^19^, in which a much greater abundance of Gypsy elements compared with Copia elements was observed. The LTRs in the *C. chinensis* genome exhibited nonrandom patterns of chromosomal distribution (Fig. 1d), and many of them were intact elements, including totals of 13,289 and 4387 intact Gypsy and Copia retrotransposons, respectively. On the basis of these intact elements, we estimated that the flood of Gypsy retrotransposition occurred approximately 1.5 million years ago (Mya), which largely occurred before the Copia burst (~2.5 Mya; Supplementary Fig. 7), suggesting that the recent influx of Gypsy rather than Copia elements in the *C. chinensis* genome is the most important reason for the observed differences in the numbers of both LTR subfamilies. We discovered some transposons (most of which are LTRs) that have genomic distributions close to (within 1.5 kb up- or downstream of a gene) or within potentially BIA genes (Supplementary Data 1), suggesting possibly creative roles of these transposons in relation to functional expression.

We estimated an average heterozygosity of 0.47% of the *C. chinensis* genome (corresponding to one heterozygous single-nucleotide polymorphism (SNP) per 212 bp) by using 4,328,940 biallelic heterozygous sites throughout the nine assembled chromosomes. The genomic distribution of these heterozygous sites was heterogeneous (Fig. 1f), with approximately 3.6% of the genome exhibiting less than one SNP locus per kilobase, while 5.1% of the genome presented more than ten SNPs per kilobase. In contrast, we did not detect any large runs of homozygous regions (>1 Mb). With the obtained consensus genome, we further inferred the occurrence of previous changes in the effective population size of *C. chinensis* using pairwise sequentially Markovian coalescent analysis. A history of weak population fluctuations for more than ten million years was observed, while a recent gradual decline in the effective population size of *C. chinensis* began approximately 100 thousand years ago, coinciding well with the inception of the last ice age (Supplementary Fig. 8).

### Phylogenomics of the Ranunculales

**Fig. 2:**
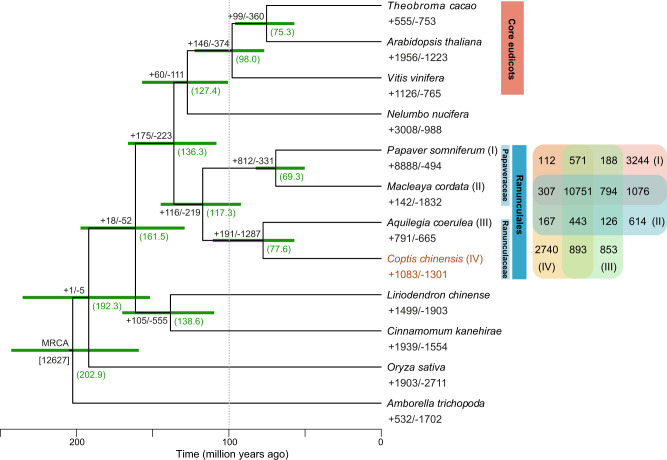
Species tree comprising 12 angiosperm species, including *Coptis chinensis*. Expansion and contraction of gene families are denoted as numbers with plus and minus signs, respectively. The green numbers in the brackets indicate the estimated divergence time of each node, and the green bars show the 95% confidence interval of divergence time, in millions of years. All the nodes are 100% bootstrap support. A Venn diagram at the side of the tree shows the sharing and specificity of gene families presented between four Ranunculales species.

Ranunculaceae are generally referred to as early diverging eudicots given that they compose the outgroup with respect to most other core eudicot lineages^20^. To investigate the details in relation to the phylogenetic placement of *C. chinensis*, we constructed phylogenetic trees based on 236 strictly single-copy orthologs that were identified by grouping orthologous protein sequences from the *C. chinensis* and 11 other flowering plant species whose genome has been fully sequenced (Supplementary Table 10). We first constructed a species tree through maximum likelihood analysis^21^ of a concatenated supermatrix of the single-copy genes. A coalescence-based analysis involving the 236 gene trees was then conducted. Topographies of these two trees were highly consistent, which preferred to cluster *C. chinensis* as a sister lineage of *Aquilegia coerulea* (Ranunculaceae), and both were closely related to two Papaveraceae species *P. somniferum* and *Macleaya cordata* (Fig. 2, Supplementary Fig. 9). These four species belong to the Ranunculales, which evolved as a sister clade to other major eudicots (or core eudicots) (Fig. 2). We further estimated the divergence times of these plants using MCMCtree^22^ with calibrations. We found that the estimated divergence time of *C. chinensis* and *A. coerulea* was ~77.6 Mya, which further diverged from the most recent common ancestor of Papaveraceae at ~117.3 Mya. Notably, plants from the Ranunculales possibly diverged from the clade including core eudicots and *Nelumbo nucifera* at approximately 120–140 Mya, consistent with the findings of Guo et al.^16^ (Fig. 2).

We compared the *C. chinensis* proteome with that of the other 11 plant species included in our phylogenomic analyses to investigate evolutionary specialization. A total of 32,181 orthologous gene families consisting of 315,336 genes were identified across these species, including a core set of 7444 gene families containing 129,109 genes shared among them, while 2397 gene families containing 8652 genes were unique to *C. chinensis* (Supplementary Fig. 10 and Supplementary Table 11). Moreover, we found a total of 10,751 gene families, including 69,027 genes that were shared among the four species belonging to the Ranunculales (Fig. 2), among which 42 gene families and 352 genes were specifically present in the Ranunculales. Interestingly, functional annotation demonstrated that these clade-specific gene family members are enriched in Gene Ontology (GO) categories such as (*S*)-norcoclaurine synthase activity (GO:0050474) and alkaloid metabolic processes (GO:0009820) (Supplementary Fig. 11), suggesting that they have possible roles related to the production of common precursors of the downstream biosynthesis of various BIA alkaloids in the Ranunculales. Moreover, gene families in relation to transporter activity (GO:0005215) and electron carrier activity (GO:0009055) are also highly enriched. Given the fact that many Ranunculales taxa are undergrowth plants in relatively high altitudes, the enrichment of genes in these two GO categories may improve their adaptability under stress exposure (e.g., absence of light, low temperature, or high salinity) and plant defense^23^. In addition, gene family evolution analysis revealed that 1083 gene families in *C. chinensis* underwent expansion, whereas 1301 gene families underwent contraction (family wide *p* value ≤ 0.01) (Fig. 2). For the expanded gene families, significantly enriched CYP genes in possibly functional pathways were observed (Supplementary Data 2).

### Ancient whole-genome duplication

**Fig. 3:**
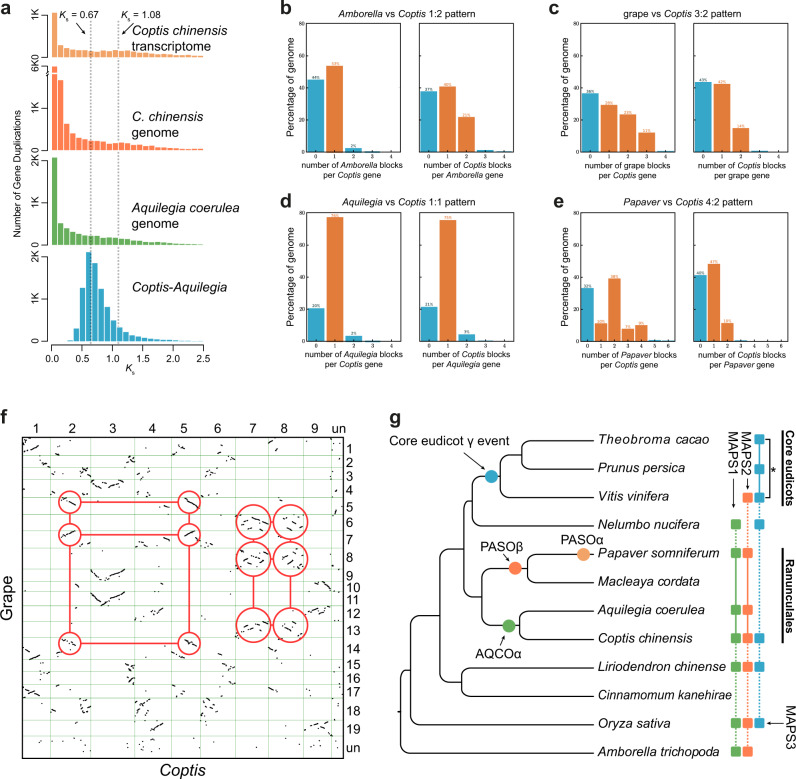
One-round ancient whole-genome duplication in *Coptis chinensis*. **a** Histogram distribution of synonymous divergence (*K*_s_) for orthologue duplicates identified within *C. chinensis* (both transcriptome and genome data were used), *A. coerulea* and between *C. chinensis* and *A. coerulea*. **b**–**e** The syntenic depth ratios between different species genome pairs. **f** Dot plots of orthologues between *C. chinensis* and grape. The red circles highlight examples of major duplication events which suggesting a 2:3 syntenic relationship. **g** The estimated whole-genome duplication (WGD) events within the Ranunculales clade and the episodic burst of gene duplications which are statistically consistent with the WGD shared by the three core eudicot species in the Multi-tAxon Paleopolyploidy Search (MAPS) analysis (marked as an asterisk in MAPS3). The taxa included in each MAPS analysis were denoted in round-corner boxes and the ingroup taxa of each analysis were linked as a solid line.

The gene age distributions and syntenic dotplots provided clearly visual evidence for an ancient whole-genome duplication (WGD) in the *C. chinensis* genome (Fig. 1g and 3, Supplementary Figs. 12–22). To infer ancient WGDs in *C. chinensis*, we first used age distribution of duplicated genes followed by a mixture model implemented in the mixtools R package to identify significant peaks of gene duplications consistent with WGDs. The mixture model identified a peak around *K*_s_ 1.08 (named as AQCOα, Fig. 3a, Supplementary Table 12). We compared it to the duplicated gene age distributions of the *A. coerulea* genome, and the *Coptis teeta* and *C. chinensis* transcriptomes. A similar peak of duplication with a median *K*_s_ around 1.08 was also found in *A. coerulea* and two *Coptis* transcriptomes (Fig. 3a, Supplementary Fig. 12 and Supplementary Table 12). This median *K*_s_ is older than the ortholog divergence of *C. chinensis* and *A. coerulea* (*K*_s_ 0.67) (Fig. 3a, Supplementary Table 13), suggesting that an ancient WGD event occurred in the ancestry of *Coptis* and *Aquilegia*. Previous studies of Papaveraceae genomes inferred a single round of ancient WGD in *M. cordata*^24^ and *P. somniferum*^16^. We used *K*_s_ plots and ortholog divergence between these genomes to assess whether the Papaveraceae WGD(s) is shared with *Coptis* and *Aquilegia*. We found the Papaveraceae WGD (PASOβ, *K*_s_ 0.72) is older than the divergence of *M. cordata* and *P. somniferum* (*K*_s_ 0.68) and predated the divergence of Ranunculaceae and Papaveraceae (*K*_s_ 0.99) (Supplementary Fig. 13 and Supplementary Table 13).

To confirm that *C. chinensis* has undergone a single round of ancient WGD, we compared the syntenic depth ratio between the *C. chinensis* and the *Amborella trichopoda* and *Vitis vinifera* genomes. We observed an overall one-to-two syntenic depth ratio between *A. trichopoda* and *C. chinensis* (Fig. 3b), which means a single *A. trichopoda* region is aligned to two *C. chinensis* blocks (Supplementary Fig. 15). Given the *A. trichopoda* genome has not experienced any WGDs after the ancestral angiosperm genome duplication event^25^, the overall one-to-two syntenic depth ratio suggests that *C. chinensis* has experienced only a single additional ancient WGD. We then compared *C. chinensis* to the *V. vinifera* genome, which was triplicated during the eudicot hexaploidy event^26^. Consistent with our hypothesis of a single WGD in *C. chinensis*, we found a two-to-three syntenic depth ratio between *C. chinensis* and *V. vinifera* (Fig. 3c). We further compared the *C. chinensis* genome to the Ranunculales genomes from *A. coerulea* and *P. somniferum*. We found a one-to-one syntenic depth ratio to *A. coerulea* (Fig. 3d), but a two-to-four ratio to *P. somniferum* (Fig. 3e). These results suggest that *P. somniferum* has likely experienced two rounds of ancient WGDs. For comparison to the *C. chinensis*, we also analyzed the syntenic depth ratios between *A. coerulea* and *P. somniferum* to *A. trichopoda* and *V. vinifera*. In *A. coerulea*, we found a two-to-one syntenic depth ratio to *A. trichopoda* (Supplementary Fig. 19), and two-to-three ratio to *V. vinifera* (Supplementary Fig. 20). In *P*. *somniferum*, we recovered a four-to-one ratio to *A. trichopoda* (Supplementary Fig. 21), and a four-to-three ratio to *V. vinifera* (Supplementary Fig. 22). Overall, our results showed syntenic evidence of a single round of ancient WGD in *C. chinensis* and an additional round of ancient WGD in *P. somniferum*.

A recent study suggested that the ancient WGD in *A. coerulea* represents the first step of the eudicot paleohexaploidy event and is shared by all eudicots^20^. Given *C. chinensis* is currently the closest related genome to *A. coerulea* and has experienced the same ancient WGD event, we used the Multi-tAxon Paleopolyploidy Search (MAPS) approach to test the hypothesis proposed by Aköz and Nordborg^20^. The MAPS algorithm uses collections of gene trees for subtrees consistent with relationships at each node in the species tree^27^. It infers shared duplications by counting the number of gene duplications shared by descendant taxa at each node and compares the actual results to null and positive simulations of WGDs. We selected three Ranunculales genomes, *C. chinensis*, *A. coerulea*, and *P. somniferum* for MAPS analyses. We also used *N. nucifera*, *L. chinense*, *Oryza sativa*, and *Amborella trichopoda* as outgroups (MAPS1, Fig. 3g). To better test whether the ancient WGD in *Coptis* is the first step of the eudicot paleohexaploidy event, we also included two alternative MAPS analyses. One retained the same species but replaced *N. nucifera* with *V. vinifera* (MAPS2, Fig. 3g). In the second alternative MAPS analysis, we used three core eudicot species (*Theobroma cacao*, *Prunus persica*, and *V. vinifera*) as ingroups and selected *N. nucifera, C. chinensis, L. chinense*, and *O. sativa* as outgroups (MAPS3, Fig. 3g). Based on the percentage of gene duplications shared by descendant taxa, and comparison to null and positive simulations of WGD, we found one episodic burst of shared gene duplication which was statistically consistent with our positive simulations of WGD at the node shared by the three core eudicot species (MAPS3, Fig. 3g, Supplementary Data 3 and 4). However, we do not find any significant gene burst at the node shared by the *N. nucifera* and Ranunculaes (MAPS1, Supplementary Data 3 and 4) or core eudicots and Ranunculaes (MAPS2, 3, Supplementary Data 3 and 4). Overall, we find no statistical support for the hypothesis suggesting *Aquilegia* experienced the first round of WGD in the eudicot paleohexaploidization.

### Genes involved in berberine biosynthesis

We identified diverse protoberberine-type alkaloids that occur predominantly in the rhizomes of *C. chinensis*, including extra high levels of berberine, followed by coptisine, palmatine, epiberberine, and jatrorrhizine (Supplementary Fig. 23). The biosynthesis and divergence of protoberberines mainly start from the central intermediate (*S*)-reticuline, which is derived from the common BIA pathway with an initial condensation of both dopamine and 4-hydroxyphenylacetaldehyde to (*S*)-norcoclaurine by (*S*)-norcoclaurine synthase (NCS)^28^ (Supplementary Fig. 24). Comparatively, berberine is relatively broadly distributed across different plant families, and its biosynthesis pathway genes have been clearly elucidated in *C. japonica* by the use of cultured cells^29,30^. We mapped all functionally characterized genes involved in the berberine biosynthesis pathway to chromosomes or unmapped contigs of the *C. chinensis* genome (Supplementary Fig. 24 and Supplementary Data 5). Notably, multiple actively expressed homologous gene copies encoding NCS (including both dioxygenase-like and pathogenesis-related 10-like protein genes), (*S*)-norcoclaurine 6-*O*-methyltransferase (6OMT), (*S*)-*N*-methylcoclaurine-3′-hydroxylase (NMCH), and (*S*)-canadine synthase (CAS) were identified (Supplementary Data 5). Some of them, including two 6OMT genes on chromosome 5 (*Cch00029942* and *Cch00029957*) and two dioxygenase-like NCS genes on chromosome 2 (*Cch00007419* and *Cch00007429*), have very close genomic distributions (~120 and 500 kb genomic interval regions, respectively), suggesting that these genes possibly originated from locally tandem duplications, that the functions of these gene pairs are possibly concerted or that these genes underwent complementary subfunctionalization. However, the overall landscape of the genomic distribution of these berberine-related genes is still scattered, leading to the failure to detect any berberine biosynthesis-related gene clusters^31^.

Increased amounts of evidence have shown that plant alkaloid accumulation is regulated by transcription factors (TFs) from the WRKY, bHLH, or AP2/ERF superfamilies^32,33^. With the assembled *C. chinensis* genome, we identified 40 WRKY, 134 bHLH, and 114 AP2/ERF gene members in total, and these genes fell into diverse subfamilies respectively (Supplementary Fig. 25 and Supplementary Data 6 and 7). By the use of cultured *C. japonica* cells, two transcriptional activators CjWRKY1 (AB267401.1) and CjbHLH1 (AB564544.1), were reported to regulate the expression of nearly all berberine biosynthetic enzyme-encoding genes^34,35^. Interestingly, we identified two actively expressed gene copies coding for each of the two TFs in the *C. chinensis* genome (Supplementary Data 5). In particular, both bHLH1 gene copies (*Cch00030529* and *Cch00027435*) revealed high sequence homology (CDSs were 96% identical to each other) but distant genomic distribution (for chromosomes 4 and 7, respectively).

### P450 genes that strongly contribute to *Coptis* alkaloid diversity

Biosynthesis pathways of other *Coptis* alkaloids besides berberine have been previously proposed (Supplementary Fig. 24) on the basis of transcriptome data in *Coptis deltoidea*^36^, *C. teeta* and *C. chinensis*^37^, as well as the reports from other Ranunculales species such as *E. californica*^38^ and *P. somniferum*^39^. For coptisine biosynthesis, the conversion of (*S*)-scoulerine to (*S*)-stylopine involves the sequential formation of two methylenedioxy bridges via cheilanthifoline synthase (CFS) and stylopine synthase (SPS). In *E. californica*, both CFS (EcCYP719A5) and SPS (EcCYP719A2 and A3) enzymes are cytochrome P450 (CYP) members belonging to the CYP719 subfamily^38^. Moreover, CAS, which catalyses the conversion of (*S*)-tetrahydrocolumbamine into (*S*)-canadine, in the berberine pathway of *C. japonica* is another CYP719 member (CjCYP719A1)^40^. The proposed pathway of epiberberine biosynthesis is also possibly related to CYP719 enzymes for catalysing (*S*)-tetrahydropalmatrubine into dihydroberberine^36,37^. Several P450 enzymes, including the (S)-*N*-methylcoclaurine-3’-hydroxylase CYP80B2^40^ and the (*S*)-corytuberine synthase (CTS) CYP80G2^41^, are also key to *Coptis* alkaloid diversification.

We investigated the overall CYP genes in the *C. chinensis* genome by using both *Arabidopsis* and rice CYPs as queries. A total of 512 best hits (e-value: 1e−05) were preliminarily found and further manually edited by removing possibly truncated or degenerated gene sequences (<300 amino acids long), resulting in a final collection of 278 putative CYP genes (Supplementary Fig. 26 and Supplementary Data 8). These genes were divided into at least 38 families attributed to ten clans, suggesting a high diversity of CYP genes throughout the *C. chinensis* genome. The number of P450 genes in *C. chinensis* is comparable to that in *Arabidopsis* (249), but less than that in rice (411) and much higher than that in *E. californica* (188)^42^ and papaya (182)^43^. Moreover, the numbers of some families significantly differed between plant species, including the CYP80, CYP82 and CYP719, which are involved in BIA biosynthesis, were more enriched in two Ranunculales taxa *C. chinensis* and *E. californica*^42^ (Supplementary Data 8).

**Fig. 4:**
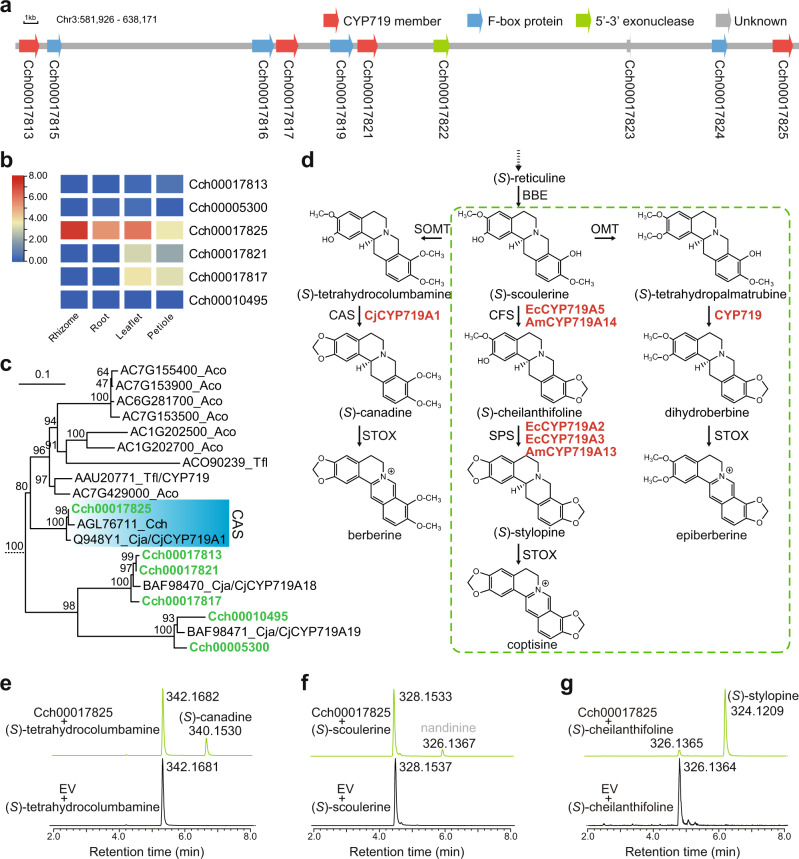
Evolution of CYP719 members in *Coptis chinensis*. **a** A gene cluster in relation to tandem duplication of CYP719 genes on chromosome 3. **b** Tissue-specific expression of CYP719 genes identified in *C. chinensis*. The expression values were log2 transformed. **c** The Ranunculaceae subclade of the CYP719 gene tree. See Supplementary Fig. 27 for a full version of the CYP719 gene tree. Gene sequences from the present study are marked in green. Aco: *Aquilegia coerulea*, Tfl: *Thalictrum flavum*, Cch: *C. chinensis*, Cja: *C. japonica*. **d** Putative branch pathways for the biosynthesis of berberine, coptisine and epiberberine. The box with the green dashed line borders indicates pathways proposed from the results of other plant species (coptisine) or suspected from the chemical structure per se (epiberberine). See Supplementary Data 5 for the abbreviations of the enzymes. The known CYP719 enzymes are denoted with a prefix of species name. Cj: *C. japonica*, Ec: *Eschscholzia californica*, Am: *Argemone mexicana*. **e**–**g** Extracted ion chromatograms showing the in vitro catalytic activity of Cch00017825 with three different substrates. In each case, enzyme-mediated activity is indicated by the green chromatograms, while those for the empty vector (EV) negative control assays are in black. The speculated product of nandinine was marked in gray. Source data underlying Fig. 4b are provided as a Source Data file.

The early diverging eudicots contain two new P450 families: CYP719 and CYP749^43^. In particular, CYP719, which possibly resulted from CYP701-related radiation in the CYP71 clan, is a Ranunculales-specific gene family, although it is also possibly present in the Aristolochiales (now is considered a synonym of Piperales in the APG IV systems)^43^. Our analysis confirmed that the identified CYP719 genes in the *C. chinensis* genome were closely related to the CYP701 family members (Supplementary Fig. 26). We further conducted comparative analysis of these CYP719 genes. Interestingly, in addition to two putative CYP719 genes, *Cch00005300* and *Cch00010495*, which have different genomic distributions on chromosomes 4 and 9, respectively, the remaining four genes are all clustered on chromosome 3 (in the region between 581,926 and 638,171) with a pattern of tandem duplication (Fig. 4a). Further analysis showed that *Cch00017813*, *Cch00005300*, and *Cch00010495* were not actively expressed, while significant expression of *Cch00017825* was present particularly in the *C. chinensis* rhizomes (Fig. 4b).

We added these genes to a recently reported CYP719 gene tree derived from 13 different plant species^44^. We found that *Cch00017825* was clustered with the CAS gene of *C. japonica CjCYP719A1* (AB026122.1)^40^ (Fig. 4c, Supplementary Fig. 27). Moreover, three genes *Cch00017813*, *Cch00017817*, and *Cch00017821* grouped with *CjCYP719A18* (ANY58145.1), while *Cch00005300* and *Cch00010495* grouped with *CjCYP719A19* (ANY58146.1). Homologous searching by using queries from encoding sequences of the previously reported CAS, CFS, and SPS genes in different plant species showed significant homology mainly presented between *Cch00017825* and the CAS gene of *C. japonica CjCYP719A1* (99.11% identical), the SPS gene *EcCYP719A2* (68.07% identical; AB126257.1)^45^ and the CFS gene of *E. californica EcCYP719A5* (64.38% identical; AB434654.1)^38^. Other comparisons revealed relatively low homology (Supplementary Data 5). The function of the *Cch00017825* gene is therefore possibly versatile, as it could serve to encode different methylenedioxy bridge-forming enzymes in the BIA biosynthesis pathway of *C. chinensis* (Fig. 4d). This phenomenon was consistent with the findings of a recent study in which no full-length transcripts encoding either CFS or SPS enzymes were detected in *C. deltoidea*^36^.

### Biochemical analysis of the targeted CYP719 genes

We employed recombinant proteins in yeast (*Saccharomyces cerevisiae*) to investigate the biochemical activity of three targeted CYP719 genes *Cch00017825*, *Cch00017817*, and *Cch00017821*. The expressions of these genes were validated using quantitative real-time polymerase chain reaction analysis (Supplementary Fig. 28 and Supplementary Table 14). Full-length cDNAs of these genes were cloned into the pESC-His vector, and the resulted constructs were further transformed into the WAT11 yeast strain with modified endogenous NADPH-CYP reductase. In vitro assays were then carried out from cultured recombinant yeast using (*S*)-tetrahydrocolumbamine, (*S*)-scoulerine, and (*S*)-cheilanthifoline as potential substrates.

Comparatively, only Cch00017825 can accept all three substrates above mentioned while the other two enzymes do not work. When (*S*)-tetrahydrocolumbamine was used as a substrate for Cch00017825, the product was found to reflect a mass reduction of [M] = 2.0152 Da and was identified as the (*S*)-canadine by comparison to an authentic standard (Fig. 4e), consistent with the generation of methylenedioxy bridge in the berberine biosynthesis pathway (Fig. 4d). When (*S*)-scoulerine was used as a substrate, a product with a mass of [M] = 326.1367 was detected (Fig. 4f). Because no available authentic standard can be used in our lab currently, it can be speculated as a product of nandinine according to a previous report^46^. A product of (*S*)-stylopine was observed by using (*S*)-cheilanthifoline as a substrate for Cch00017825 (Fig. 4g), suggesting the potential function of *Cch00017825* encoding SPS.

### Functional diversity of methyltransferase in *Coptis*

Methyltransferases (MTs), including those involved in both *O*- and *N*-methylations, represent another important enzyme group for the evolution and diversification of BIA alkaloids in plants^47^. We characterized all known MT genes in the assembled *C. chinensis* genome, including two gene copies encoding the 6OMT enzymes mentioned above and the genes encoding (*S*)-norcoclaurine 7-*O*-methyltransferase (7OMT), (*S*)-coclaurine *N*-methyltransferase (CNMT), (*S*)-3′-hydroxy-*N*-methylcoclaurine 4′-*O*-methyltransferase (4′OMT), (*S*)-scoulerine 9-*O*-methyltransferase (SOMT), and columbamine *O*-methyltransferase (CoOMT) (Supplementary Data 5). Among these enzymes, CoOMT is a key enzyme for catalysing the conversion of columbamine to palmatine^48^. 7OMT is a possible enzyme for converting (*S*)-norcoclaurine into isococlaurine in the putative biosynthesis pathway of jatrorrhizine, which contains an unusual 7-*O*-methylation pattern^37^. Grouping of the identified OMT genes in the *C. chinensis* genome with other reported genes from both *C. japonica* and *C. teeta* revealed a clear relationship except for 6OMT genes, which was in two respective clusters (6OMT1 and 6OMT2) (Supplementary Fig. 29).

## Discussion

Given the importance of Ranunculales in terms of both medicinal and phylogenetic aspects, the high-quality chromosomal-scale assembly of the *C. chinensis* genome further enriched our understanding of the genomic landscapes of early diverging eudicot lineages. The *C. chinensis* genome is comparable to those recently reported in some early evolving angiosperms, such as *L. chinense*^18^, stout camphor tree^17^, water lily^49^, and sacred lotus^50^. Benefiting from the robust assembly, we can determine the detailed genomic landscape of *C. chinensis*, including its abundance of repetitive elements, which cover 62.5% of the assembly (with an extra high presence of Gypsy retrotransposons representing 35.5% of the *C. chinensis* genome) (Supplementary Table 9). Our phylogenomic analysis clearly reveals an early-evolving eudicot placement for *C. chinensis*, and its sister relationship to *A. coerulea* (Ranunculaceae) (Fig. 2 and Supplementary Fig. 9). Given the increasing number of genome sequences reported for early-evolving flowering plant species, the phylogenetic relationships between the taxa at the base of angiosperms have become increasingly clear. Among them, *C. chinensis* and Ranunculaceae represent a key evolutionary step linking core eudicots and other early diverging angiosperms, such as magnoliids and monocots, giving rise to the evolution of various traits, such as the original possession of triaperturate pollens^11^.

We inferred and placed an ancient WGD in *Coptis* by incorporating *K*_s_ plots, ortholog divergences, synteny, and phylogenomic analyses. Our inferences are consistent with previous studies from the 1KP project^51^ and two *Aquilegia* genomes^14,20^. By comparing synteny between *C. chinensis* and other Ranunculales genomes, we confirmed an additional round of ancient WGD in *P. somniferum* that was previously missed^16^. Previous studies found a single WGD in *M. cordata*^24^ and *P. somniferum*^16^. However, a recent study based on multiple transcriptomes found evidence for two rounds of ancient WGD in *P. somniferum*^51^. By comparing *P. somniferum* to the *C. chinensis* genome, we found a four-to-two syntenic depth ratio. Along with the 1KP study, this result provides clear evidence of two rounds of ancient WGD in *P. somniferum*. The *K*_s_ plots and ortholog divergences further suggest this older round of ancient WGD predates the divergence of *M. cordata* and *P. somniferum* but it is not shared with the Ranunculaceae (Supplementary Tables 12 and 13). This ancient WGD in *P. somniferum* was overlooked by Guo et al.^16^, possibly due to the quality of the genome assembly or the lack of closely related genomes for comparison. These results highlighted the value of the high-quality *C. chinensis* genome in comparative studies to Ranunculales and other Eudicots.

*A. coerulea* represents the first genome of Ranunculaceae^13^. A recent study suggests an ancient WGD inferred in *A. coerulea* represents the first step of the eudicot paleohexaploidy event^20^. Based on this hypothesis, early diverging eudicot lineages that are not core eudicots, such as Ranunculales and Proteales, should also share this ancient polyploidy. This result contradicted previous analyses which found no evidence of this shared WGD in the *N. nucifera* genome^52,53^ and many eudicot transcriptomes^51^. Using the MAPS phylogenomic approach, we tested this hypothesis with *N. nucifera*, *C. chinensis*, *A. coerulea*, and other genomes. We found strong evidence for the eudicot paleohexaploidy event shared by *T. cacao*, *P. persica*, and *V. vinifera* (MAPS3 in Fig. 3g, Supplementary Data 3 and 4). However, we found no statistical support for an ancient WGD shared by Ranunculales, Proteales, and core eudicots. Inconsistent with the hypothesis by Aköz and Nordborg^20^, our results are inline with other recent studies on ancient WGDs in Ranunculales^19,52,54^. However, our hypothesis testing is based on the current phylogenetic relationship between Ranunculales, Proteales, and other eudicot lineages. Given the short internal branches^51^, and the rapid diversification following the paleohexaploidy event^55^, future studies on better resolving the eudicot phylogeny and testing this hypothesis is still needed. Overall, we provide clear evidence for a single ancient WGD shared by *Coptis* and *Aquilegia* and our ancient WGD analyses are largely consistent with previous genomic inferences.

The diversification of plant cytochrome P450 genes is one of the primary driving forces of phytochemical diversity^54^. In the BIA biosynthesis pathways of *C. chinensis*, CYP-mediated methylenedioxy bridge formation (CYP719 members), hydroxylation (CYP80B) and phenol coupling reactions (CYP80G) greatly contributed to the final diversity of protoberberine-type alkaloids. The Ranunculales clade-specific evolution of the CYP719 family is remarkable. Currently, nearly all methylenedioxy-bridge formation reactions in isoquinoline alkaloid biosynthesis are catalyzed by the CYP719A subfamily members^54^. Our analysis revealed a pattern of tandem duplications for driving the quick formation and diversification of CYP719A members (Fig. 4a). A similar pattern was observed for the evolution of some other CYP family members (e.g., CYP81 and CYP82; Supplementary Fig. 26). Moreover, we failed to detect any BIA gene-related BGCs in the *C. chinensis* genome. This is consistent with the opinion that a few important metabolic clusters involve only tandem repeated arrays within a single gene family^56^, rather than BGCs which refer to genomic loci that encode genes for a minimum of three different types of biosynthetic reactions (i.e., genes encoding functionally different (sub)classes of enzymes^31^). Indeed, many plant biosynthetic pathway genes are not clustered together, such as those of anthocyanins^57^, and some plant pathway genes can occur within more than one BGC in a particular genome^58^. Alternatively, given the potentially ancient evolution of the berberine-related pathways, which are widespread among taxa in more than one plant family, the more specialized and derived long-branch pathways, such as the noscapine and morphinan branches in opium poppy^16^, are more likely to evolve in accordance with BGC pattern.

Our results revealed potentially versatile functions of a key berberine pathway gene *Cch00017825* (encoding both CAS and SPS, Fig. 4e, g). Functional diversification of the main BIA genes seems to be a universal phenomenon. In California poppy (*E. californica*), CYP719A5 has cheilanthifoline synthase activity^38^, and both CYP719A2 and CYP719A3 have stylopine synthase activity^45^. Enzymatic analysis further showed that CYP719A3 had high affinity for three similar substrates, (*R,S*)-cheilanthifoline, (*S*)-scoulerine, and (*S*)-tetrahydrocolumbamine, suggesting that this enzyme has potentially diverse functional roles. Similarly, the CYP719A13 enzyme identified in the Mexican prickly poppy (*Argemone mexicana*) revealed function of stylopine synthase activity^46^. Moreover, CYP719A13 can further convert (*S*)-tetrahydrocolumbamine to (*S*)-canadine. For methyltransferases, the characterized OMTs of BIAs can also display diverse functions in different plant species. For example, 6OMT in both *C. japonica* and *C. chinensis* and 7OMT in *C. teeta* are able to catalyze reactions involving various substrates^37^. The evolutionary forces driving the functional diversity of BIA enzymes are not yet fully understood. Given the potentially monophyletic origin and repeated evolution of BIA genes, the functions of ancestral BIA enzymes are possibly highly unrestrained and perhaps involve the catalysis of a large number of substrates with relatively low efficiency^47^.

In conclusion, our study of an important evolutionary lineage with remarkable medicinal applications provides critical information that adds to the limited genomic resources of early diverging eudicots. We found a single round of ancient WGD in *C. chinensis* that predated the divergence of *Aquilegia* and *Coptis*. This provides information for future studies on resolving the origin of eudicots. Given the medicinal values of protoberberine-type alkaloids, our analysis will further contribute to the understanding of the complex pathways driving diversification of plant BIA alkaloids, including the specific evolution and functional versatility of cytochrome P450 genes.

## Methods

### Plant materials

In China, the CRs of *C. chinensis*, *C. deltoidea* and *C. teeta* are all used in TCM. Among these, *C. chinensis* is the most important one and has a relatively widely natural distribution, whereas *C. teeta* is an endangered species. We therefore choose *C. chinensis* for our genome sequencing. Five-year-old adult *C. chinensis* plants were collected from Lichuan, Hubei Province, a popular genuine production area of the Chinese goldthread. Fresh leaves were used for isolating total genomic DNA using the conventional cetyltrimethylammonium bromide method.

### Genome sequencing

For the Oxford Nanopore library preparation, a total of 10 µg of high-molecular-weight DNA was subjected to random fragment shearing using a Megaruptor (Diagenode, NJ, USA). Agencourt Ampure XP beads (Beckman, Indiana, USA) were used for excluding small DNA fragments. The bead size-recovered DNA was repaired and end-modified using the NEBNExt Ultra II End-repair/dA tail module, and adapters were added using Blunt/TA Ligase Master Mix (NEB, Beverly, MA). Then, further purifying using Ampure XP beads and ABB wash buffer (ONT, Oxford, UK) was performed. The resulted library was combined with RBF (ONT, Oxford, UK) and loaded onto primed R9.4 Spot-On Flow cells (Oxford Nanopore Technologies) for sequencing on a PromethION high-throughput sequencer. Base calling analysis of the unprocessed data was performed using Oxford Nanopore Albacore software (v2.1.3).

We also generated ~100× Illumina short reads on the HiSeq2000 platform (Illumina, San Diego, CA, USA) with insert sizes of 180 and 300 bp for polishing the genome assembly. Raw sequencing data were processed using FastQC (www.bioinformatics.babraham.ac.uk/projects/fastqc) for detecting poor quality reads and over-represented sequences. We prepared the Hi-C library following a standard procedure^59^. In situ cross-lined DNA from approximately 700 ng of high-quality genomic DNA was extracted and digested with a restriction enzyme. The sticky ends of the digested DNA were labeled by biotin and ligated to each other randomly to form chimeric junctions. These biotinylated DNA fragments were further enriched and sheared to prepare the library for sequencing on the Illumina platform.

### Genome size estimation

We first estimated the size of the *C. chinensis* genome using a *k*-mer (*k* = 19) analysis-based approach together with Illumina paired-end short reads. The software Jellyfish (v2.1.4)^60^ was used for counting *k*-mers in the DNA sample, and the software GenomeScope (v1.0)^61^ was used for estimating the genome size, resulting in a predicted genome size of ~1.02 Gb. A second determination of the genome size was based on flow cytometry. The 2 C DNA content of *C. chinensis* was determined on a BD Accuri^TM^ C6 flow cytometer (BD Biosciences, San Jose, CA, USA) at 211,686.82 and compared with that of diploid kiwifruit at 135,732.57 (1.53 pg/2 C)^62^ and *Chrysanthemum nankingense* at 600,764.85 (6.63 pg/2 C)^63^ (Supplementary Fig. 2), yielding an approximate nuclear DNA amount of 2.36 pg/2 C for *C. chinensis*, suggesting a genome size of approximately 1.15 Gb (1 pg = 0.978 Gb) for the haplotype.

### RNA sequencing

The roots, rhizomes and the main aerial tissues of leaflets and petioles of *C. chinensis* were used for RNA-sequencing (Supplementary Fig. 1). The total RNAs of these tissues were extracted using a HiPure Plant RNA Kit (Magen, Guangzhou, China) and further purified using a NEBNext Ultra RNA Library Prep Kit for Illumina (New England Biolabs, Inc.). Approximately, 2 μg of RNA for each sample was prepared to construct the RNA-seq libraries using an Illumina TruSeq library Stranded mRNA Prep Kit, after which the RNA was sequenced on the Illumina platform.

### Genome assembly and quality assessment

We used a hybrid strategy to assembly the genome of *C. chinensis*. Briefly, we used Canu (v1.6)^64^ to correct the Nanopore long reads and then used SMARTdenovo (v1.5) (https://github.com/ruanjue/smartdenovo) to assemble them with parameters of -p jvh -k 17 -J 500 -t 32 -c 1. After finishing the initial assembly, two rounds of polishing were conducted, including using Racon (v1.3.2)^65^ to directly polish the assembly only produced from Nanopore long reads, and using Pilon (v1.22)^66^ for iterative polishing in which adapter-trimmed paired-end Illumina reads were aligned with the raw assembly. Given the high heterozygosity presented in some genomic regions which can lead to the assembly of regional duplication rather than consolidation into allelic variants, we further used Purge Haplotigs^67^ to improve the resulted assembly by removing duplications and reassigning allelic contigs. The quality of the assembly was assessed via BWA-MEM algorithm in BWA (0.7.17)^68^ by aligning the Illumina short reads and the assembled transcripts to the genome. We further performed BUSCO (v2.0) assessments on the assembly.

### Gene prediction and functional annotation

Transcripts from the *C. chinensis* tissues were assembled de novo using Trinity (v2.3.3)^69^. Proteomes of 11 representative plant species (Supplementary Table 10) were downloaded and used as homologous references. The Maker package (v2.31.10) (https://www.yandell-lab.org/software/maker.html) was used to predict the putative protein-coding genes in *C. chinensis*. Using Augustus (v3.2.1)^70^, we also performed de novo predictions of the obtained gene structures. The rRNAs were predicted using RNAmmer (v1.2)^71^, tRNAs were predicted using tRNAscan-SE (v1.23)^72^, and ncRNAs were identified using the Perl program Rfam_scan.pl (v1.0.4) via inner calling using Infernal (v1.1.1)^73^.

We carried out functional annotations of the protein-coding genes using BLASTP (v0.7.9; *e*-value cut-off 1e−05) searches against entries in both the NCBI nr and UniProt databases. Searches for gene sequence motifs and structural domains were performed using InterProScan (v5.33)^74^. The GO terms of the genes were obtained from the corresponding InterPro or Pfam entry. Pathway reconstruction was performed using KOBAS (v2.0)^75^ and the KEGG database.

### Annotations of repetitive sequences

We used RepeatModeler (v1.0.4) (https://github.com/rmhubler/RepeatModeler) to identify repetitive sequences in the *C. chinensis* genome de novo. We further used RepeatMasker (v4.0.5) (http://www.repeatmasker.org/) analysis to search for known repetitive sequences using a cross-match program with a Repbase-derived RepeatMasker library and the de novo repetitive sequences constructed by RepeatModeler. We used the equation *t* = *K*/*2r* to estimate the integration times (*t*) of intact LTRs, where *K* is the number of nucleotide substitutions per site between each LTR pair and *r* is the nucleotide substitution rate, which was set to 1 × 10^−8^ substitutions per site per year^76^.

### Genome heterozygosity and demography

Using BWA–MEM algorithm, we aligned the paired-end Illumina reads of *C. chinensis* to the assembled genome. Only reads with a high mapping score (greater than 20) were retained and the bam files were sorted via PicardTools (v1.95; http://broadinstitute.github.io/). Variable sites were identified using SAMtools^77^ (v1.11), and consensus sequences were generated using BCFtools^78^ (v1.7). We inferred the past effective population size of *C. chinensis* using the PSMC program^79^. The results of the demographic modeling were scaled using an estimated synonymous substitutions per site per year of ~0.5 × 10^−8^ for Ranunculales^16^ and a generation time of 6 years.

### Phylogenomic analysis

The amino acid sequences of single-copy genes from 12 species (Supplementary Table 10) were aligned using MUSCLE (v3.8.31) (https://www.ebi.ac.uk/Tools/msa/muscle/). Following alignment, the proteins were reverse-transcribed into their coding sequence, and RAxML (v8.2.10)^21^ was used to construct a phylogenetic tree according to the model of PROTGAMMAWAG and 500 bootstrap replicates. A phylogenetic tree of each single-copy gene was further constructed to infer a consensus species tree using ASTRAL-III^80^. We used the Bayesian relaxed molecular clock method to estimate species divergence times. The program MCMCtree (v4.0) within the PAML package (v4.8)^22^ was used, and the divergence times of *M. cordata*–*P. somniferum* (~44–82 Mya) and monocots–dicots (~130–240 Mya) were used for calibration.

Protein-coding genes from 12 plant species (Supplementary Table 10) were used to perform an all-against-all comparison using BLASTP (v0.7.9; e-value cut-off 1e−05), and the orthologous gene family clusters between these plant species were identified by the OrthoMCL (v2.0.5)^81^ program. Gene family expansion and loss were inferred using CAFÉ (v4.1)^82^, with an input species tree constructed from the single-copy orthologs.

### Investigation of whole-genome duplication

We used the DupPipe pipeline to construct gene families and estimate the age distribution of gene duplications^83,84^. We estimated synonymous divergence (*K*_s_) using PAML in conjunction with the F3X4 model^22^ for each node in the gene-family phylogenies. We then used a mixture model implemented in the mixtools R package (https://CRAN.R-project.org/package=mixtools) to identify significant peaks that are consistent with a potential WGD event, and significant peaks were identified using the likelihood ratio test of the boot.comp function of the package^85^. The genomic collinearity blocks for intra- and interspecies comparisons of *Coptis* were identified by MCScan^86^. We performed all-against-all LAST^87^ and chained the LAST hits with a distance cut-off of ten genes, requiring at least five gene pairs per synteny block. The syntenic “depth” function implemented in MCScan was applied to estimate the duplication history in the respective genomes. Genomic synteny was ultimately visualized by the Python version of MCScan (https://github.com/tanghaibao/jcvi/wiki/MCscan-(Python-version)).

### MAPS analyses of WGDs from genomes of multiple species

To test the hypothesis of the placement of the eudicot hexaploidy, the MAPS tool^27,88^ was applied. Briefly, orthologous groups for selected species were obtained from Orthofinder^89^. The phylogenetic trees for all gene families were constructed by PASTA^90^ and further analyzed by the MAPS program.

Both null and positive simulations were performed to compare with the observed number of duplications at each node. For null simulations, the gene birth rate (*λ*) and death rate (*μ*) for the selected six species were estimated with WGDgc^91^. Gene count data of each gene family of the six species were obtained from Orthofinder. The estimated parameters were configured in MAPS and the gene trees were then simulated within the species tree using the GuestTreeGen program from GenPhyloData^92^. For each species tree, we simulated 3000 gene trees with at least one tip per species according to the settings in the 1KP project^51^. We randomly resampled 1000 trees without replacement from the total pool of gene trees 100 times to provide a measure of uncertainty on the percentage of subtrees at each node. A Fisher’s exact test was used to identify locations with significant increases in gene duplication compared with a null simulation.

For positive simulations, we simulated gene trees using the same methods described above. However, we incorporated WGDs at the location in the MAPS phylogeny with significantly larger numbers of gene duplications compared to the null simulation. We allowed at least 20% of the genes to be retained following the simulated WGD to account for biased gene retention and loss.

### Metabolomic analysis

The contents of alkaloids from roots, rhizomes, leaflets, and petioles were investigated using a Waters 2695–2998 high-performance liquid chromatography system (Waters, Milford, MA, USA) and a previous procedure was referenced^37^. Briefly, 0.2 g of dried powder of each sample was used to extract bioactive components with hydrochloric acid-methanol mixed liquor (1:100, v/v). Chromatographic separation was performed with a mobile phase consisting of acetonitrile and 0.05 mol/L potassium dihydrogen phosphate solution (50:50, v/v), both of which contained 0.1% sodium lauryl sulfate. Detection was performed at 345 nm. Standards of berberine, coptisine, jatrorrhizine, palmatine, and epiberberine were purchased from National Drug Reference Standards (http://aoc.nifdc.org.cn/sell/home/index.html).

### Gene family analysis

The arbitrary definition of a metabolic gene cluster requires that the cluster contains genes for at least three different types of enzymes^93^. BCGs were detected using the online tool plantiSMASH (http://plantismash.secondarymetabolites.org/). Genes in relation to berberine biosynthesis reported in *C. japonica* were used as queries for searching the corresponding copies in the *C. chinensis* genome, with an e-value of 1e-5. The sequences of the *A. thaliana* and rice CYP genes were downloaded (http://drnelson.uthsc.edu/P450seqs.dbs.html) and used as queries to search for homologs and conserved domains (PF00067) against the *C. chinensis* genome. Further manual validation with the SMART tool (http://smart.embl-heidelberg.de) was conducted, and genes encoding protein sequences < 300 amino acids long were removed. To search for genes encoding methyltransferase in the *C. chinensis* genome, homologs from *C. japonica* (D29811.1, D29809.1, Q8H9A8.1, and D29812.1) and *C. teeta* (MH165877.1 and MH165874.1) were used as queries for searching via BLAST. Multiple sequence alignment of gene families was conducted using ClustalW, and a neighbor-joining tree was constructed with MEGAX (10.1.8)^94^.

### Heterologous expression in yeast and in vitro activity assay

We followed the method recently reported in *Salvia* for our biochemical analysis^95^. Briefly, the epitope-tagged pESC-His vectors carrying CYP719 genes were each transformed into the yeast strain WAT11. Strains with empty pESC-His vector was used as a control. The strain cells were centrifuged and resuspended twice in respective TEK and TESB buffers, and were then broken up by a cryogenic homogenizer. Further centrifugation was conducted for precipitation of microsome pellets. For in vitro activity assays, 0.5 mg resuspended microsomal protein and 100 μM of a potential substrate were included in a 500 μL reaction system for incubation of possible products. Alkaloids from catalytic products were identified using an ultra-high performance liquid chromatography-quadrupole time-of-flight mass spectrometry system (Waters, Milford, MA, USA) with elution buffers consisting of 0.1% aqueous formic acid and 0.1% formic acid in acetonitrile. MS analysis was conducted on a Waters Xevo G2-S QTOFmass spectrometer equipped with electrospray ionization. The acquisition of data was controlled by Waters MassLynx software (v4.1).

### Reporting summary

Further information on research design is available in the Nature Research Reporting Summary linked to this article.

## Supplementary information

Supplementary Information

Supplementary Data

Description of Additional Supplementary Files

Reporting Summary

## Data Availability

The data supporting the findings of this work are available within the paper and its Supplementary Information files. A reporting summary for this article is available as a Supplementary Information file. The datasets and plant materials generated and analyzed during the current study are available from the corresponding author upon request. The raw sequence reads and genome assembly have been deposited in NCBI under the BioProject accession number PRJNA662860 with BioSample accessions SAMN18434929-SAMN18434940. The databases of KEGG (http://www.genome.jp/kegg/), Swissprot and TrEMBL (http://www.uniprot.org/), and InterPro (https://www.ebi.ac.uk/interpro/) are used for data analyses. Source data are provided with this paper.

## Source data

Source Data
