# Supplementary material for: Analysis of the Coptis chinensis genome reveals the diversification of protoberberine-type alkaloids
Source: Nat Commun. 2021 Jun 2;12:3276. doi: 10.1038/s41467-021-23611-0 (PMC8172641; doi:10.1038/s41467-021-23611-0)
Supplement: Supplementary file 1 — Supplementary Information [file 41467_2021_23611_MOESM1_ESM.pdf]

Analysis of the *Coptis chinensis* genome reveals the diversification of protoberberine-type alkaloids

Liu *et al*.

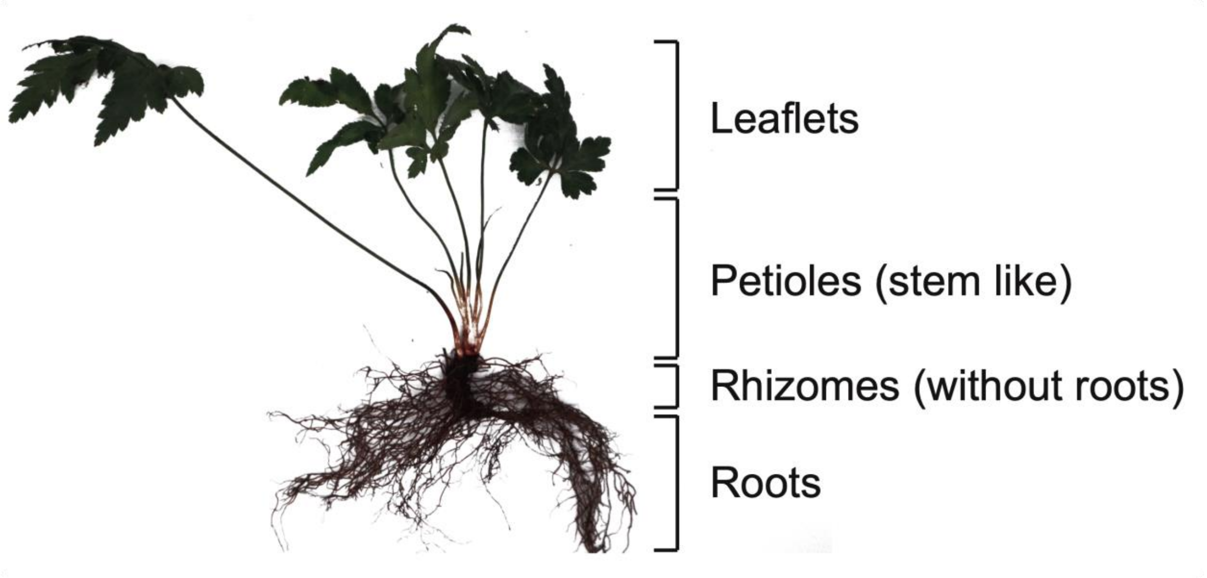

Supplementary Fig. 1. A plant of *Coptis chinensis*. It was collected from Lichuan, Hubei Province, China. Four tissues were marked for both transcriptomic and metabolic analyses.

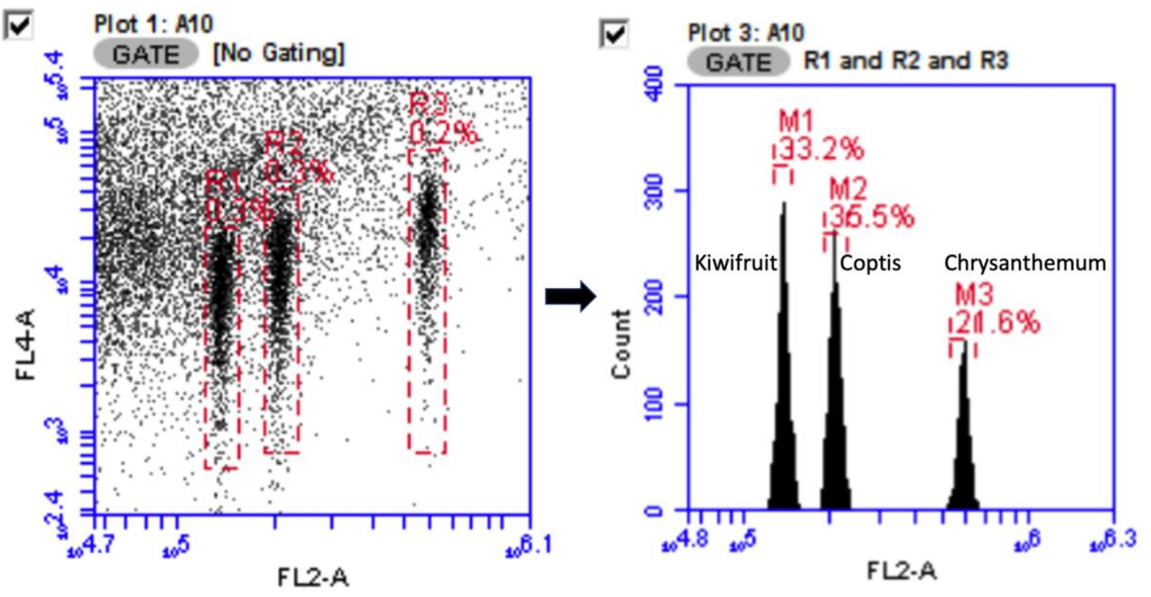

Supplementary Fig. 2. Estimate of the genome size of *Coptis chinensis* on a BD Accuri^{TM} C6 flow cytometry. Both diploid kiwifruit (*Actinidia chinensis*) and *Chrysanthemum nankingense* were used as inner standards. On an FL2 vs FL4 plot, nuclear events cluster for three samples (left) and gating on these events with FL2 fluorescence shows clear peaks corresponding to species genome size (right).

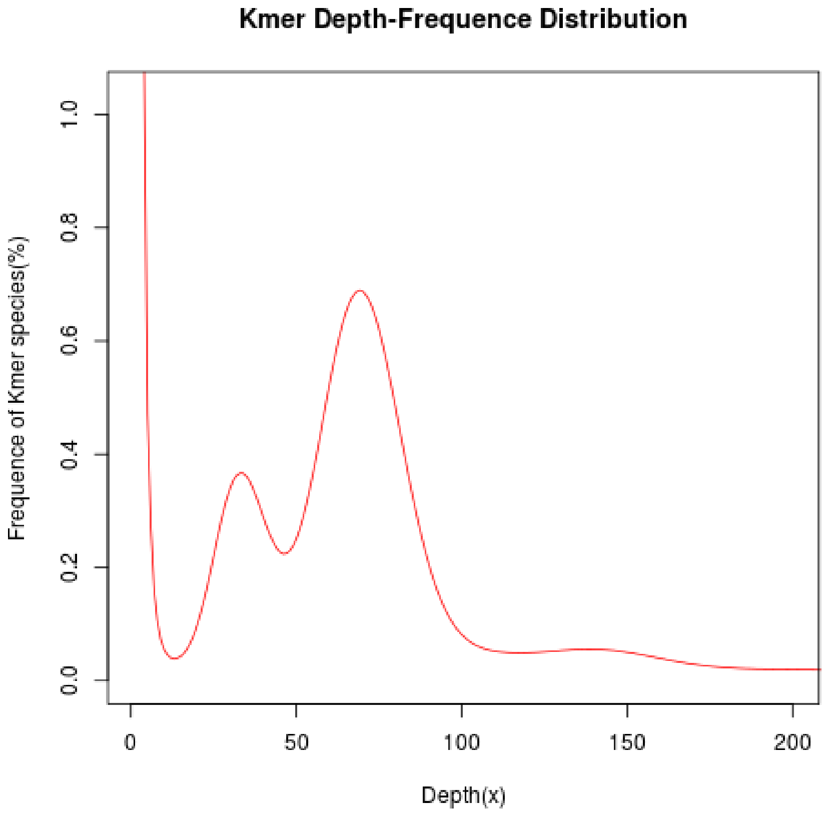

Supplementary Fig. 3. 19-mer depth distribution of the *Coptis chinensis* genome sequencing reads. Two peaks were observed indicating heterozygosity in this species.

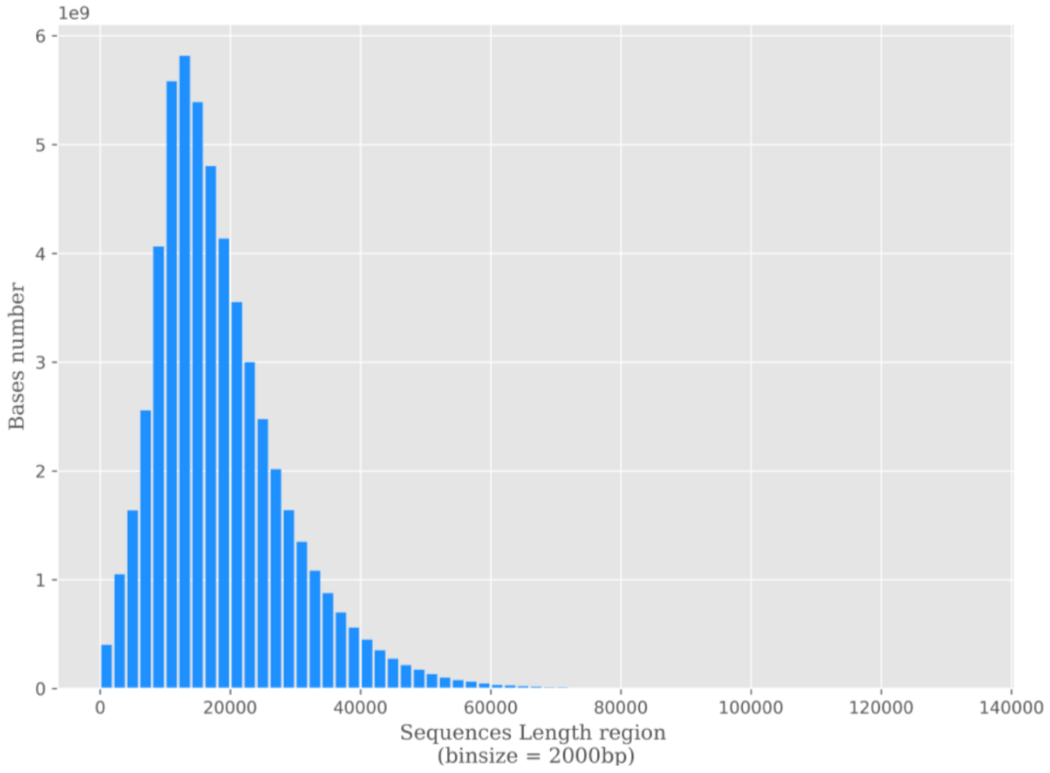

Supplementary Fig. 4. Length distribution of nanopore long reads produced from the *Coptis chinensis* sample.

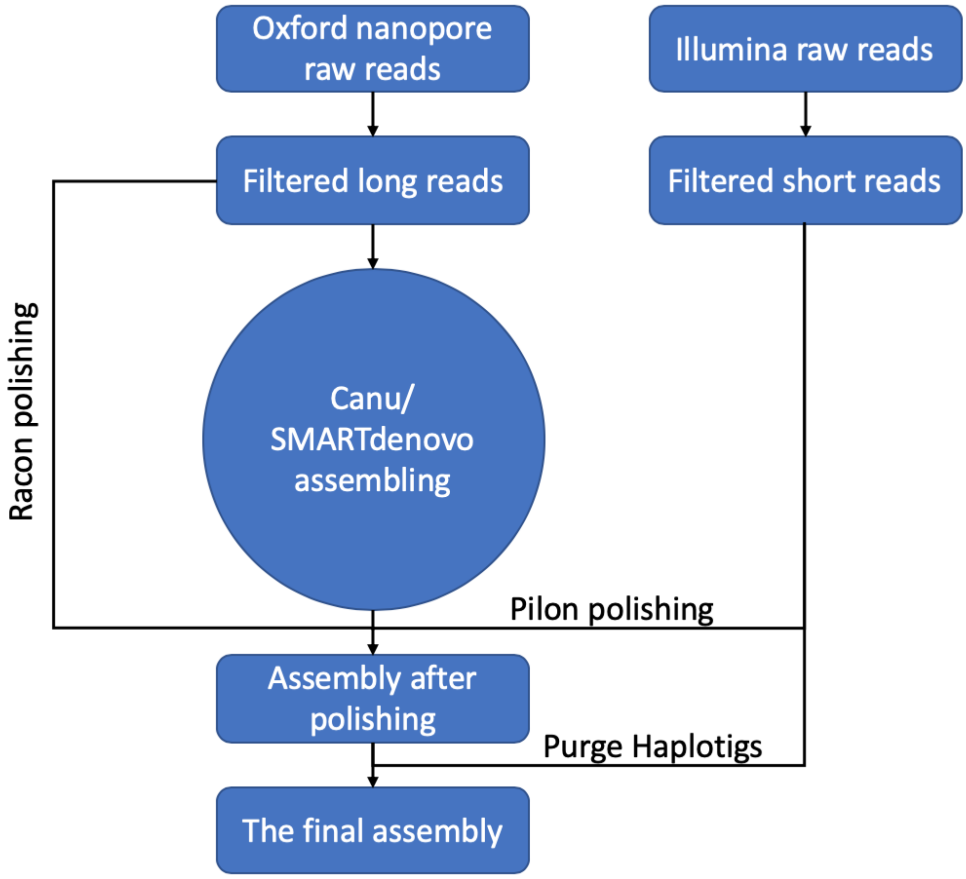

Supplementary Fig. 5. Genome assembly flowchart demonstrating assembly polishing and data integration.

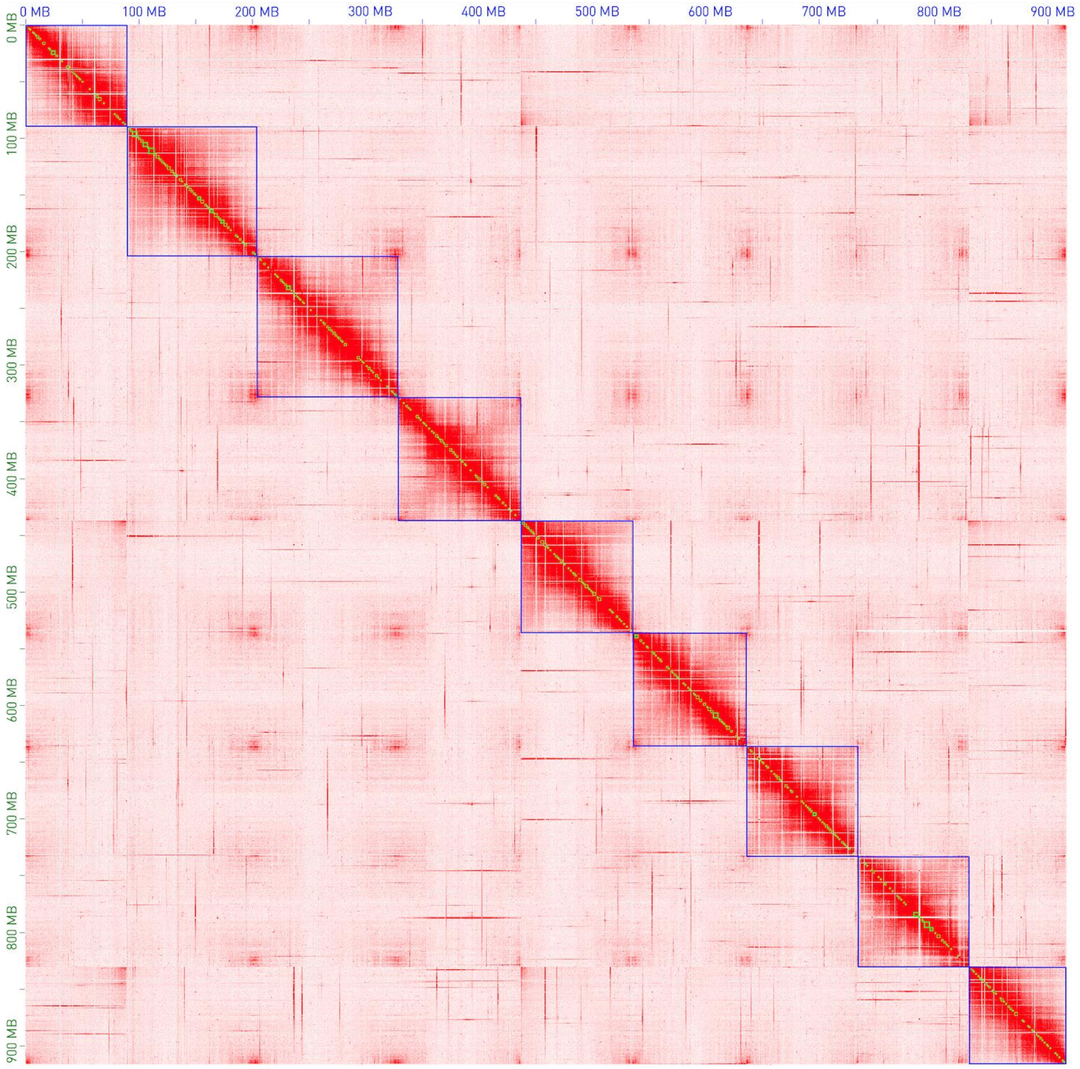

Supplementary Fig. 6. The Hi-C map of *Coptis chinensis* genome assembly.

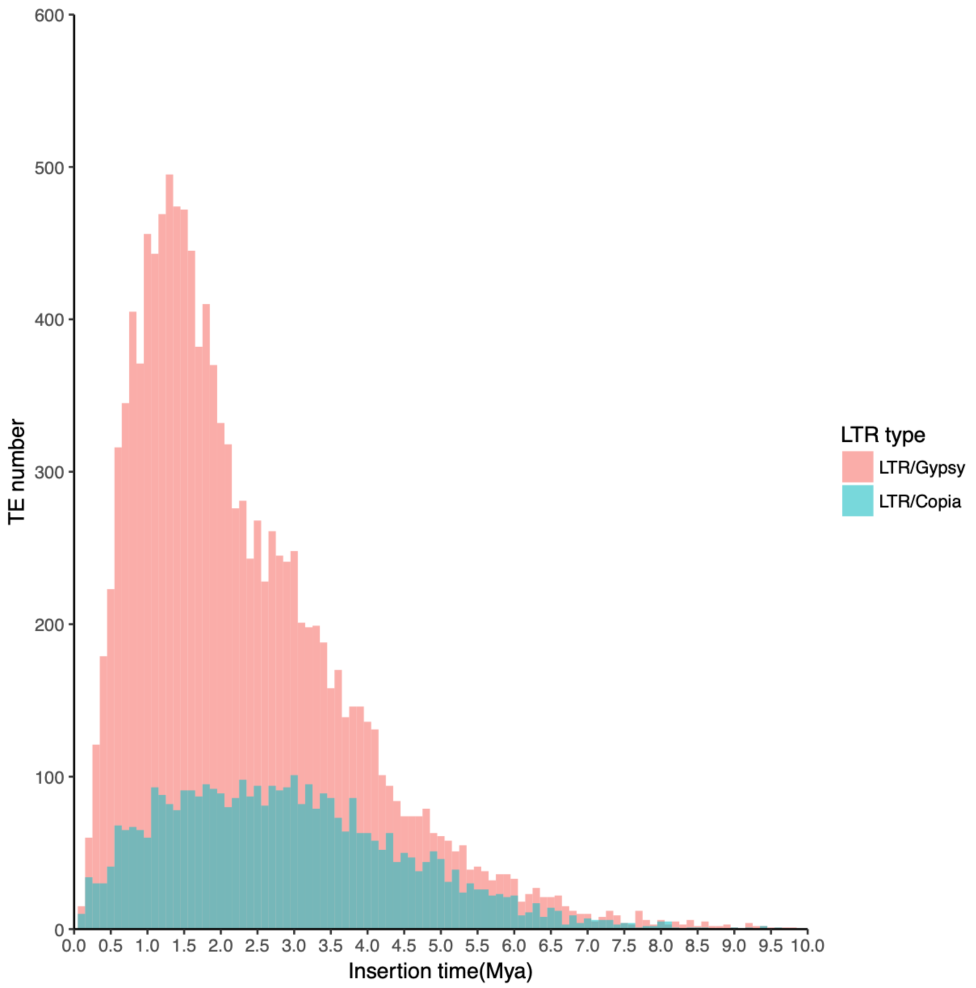

Supplementary Fig. 7. Insertion burst of Gypsy and Copia retrotransposons in the *Coptis chinensis* genome. TE, transposable element; LTR, long terminal repeat; Mya, million years ago.

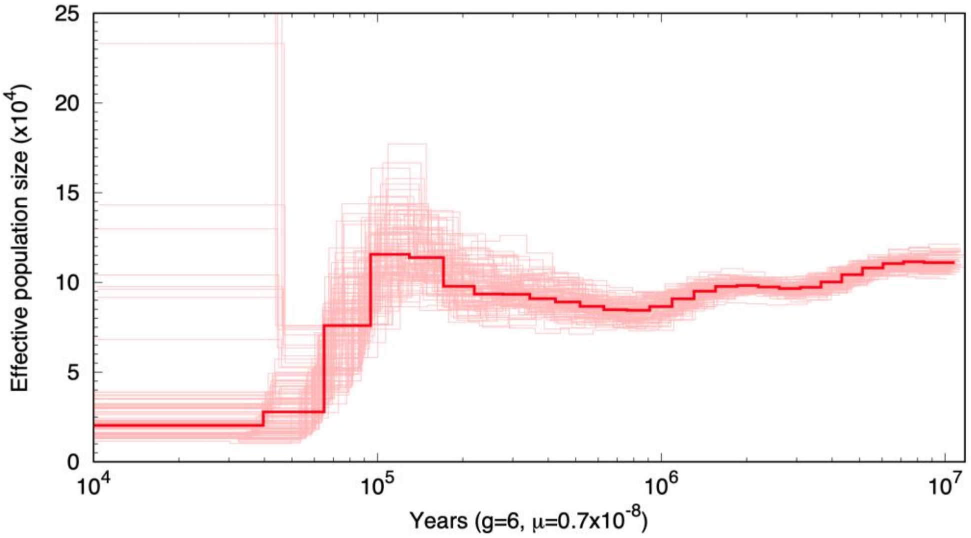

Supplementary Fig. 8. The history of effective population size of *Coptis chinensis* was inferred using the pairwise sequentially Markovian coalescent analysis.

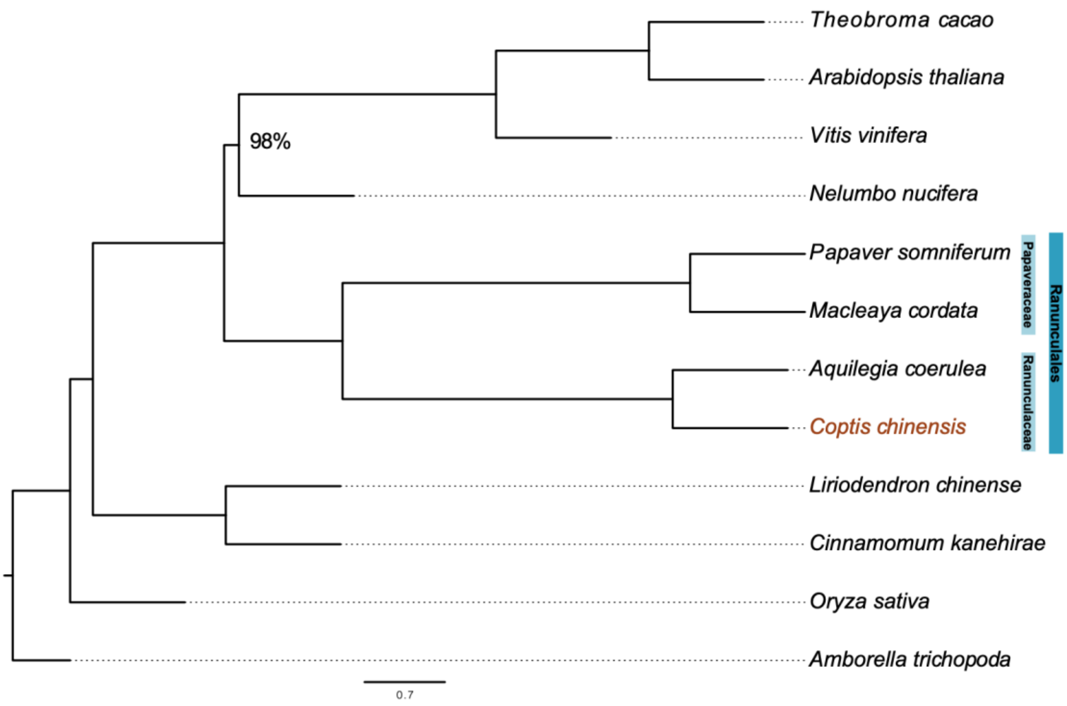

Supplementary Fig. 9. A species tree of 12 plant species on the basis of the coalescence of gene trees from 236 single copy orthologues using ASTRAL. All nodes with 100% support except denoted.

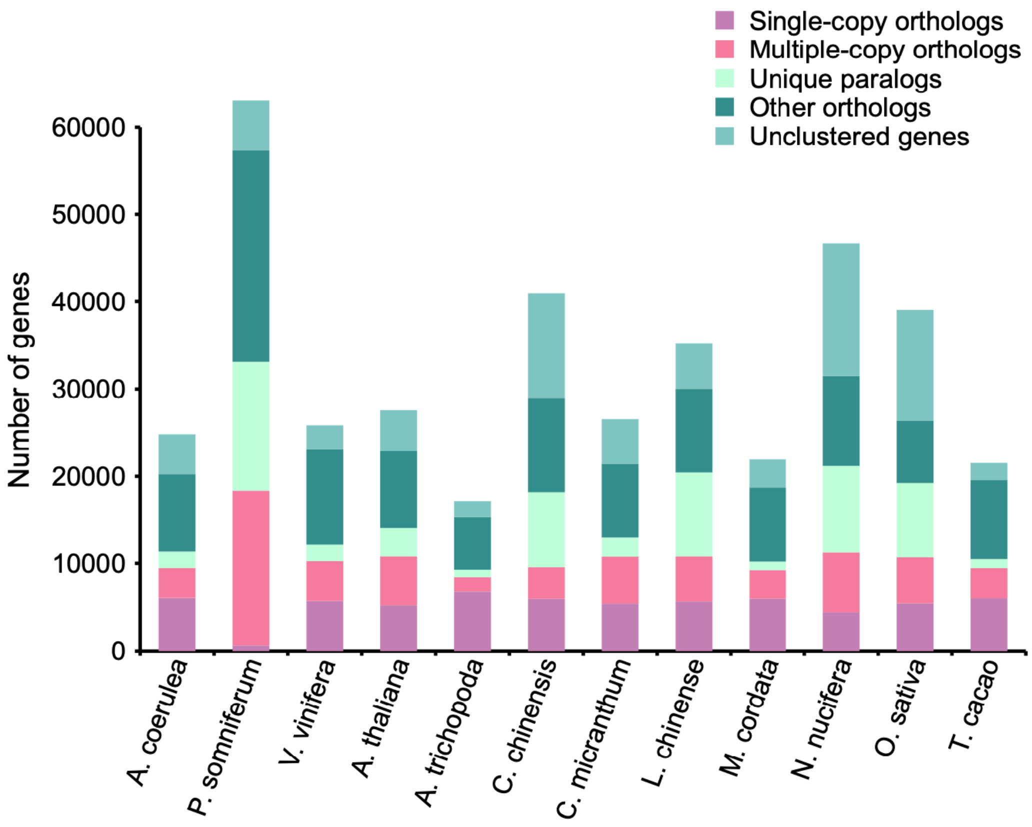

Supplementary Fig. 10. Distribution of genes and gene families of plant species we investigated. Source data are provided as a Source Data file.

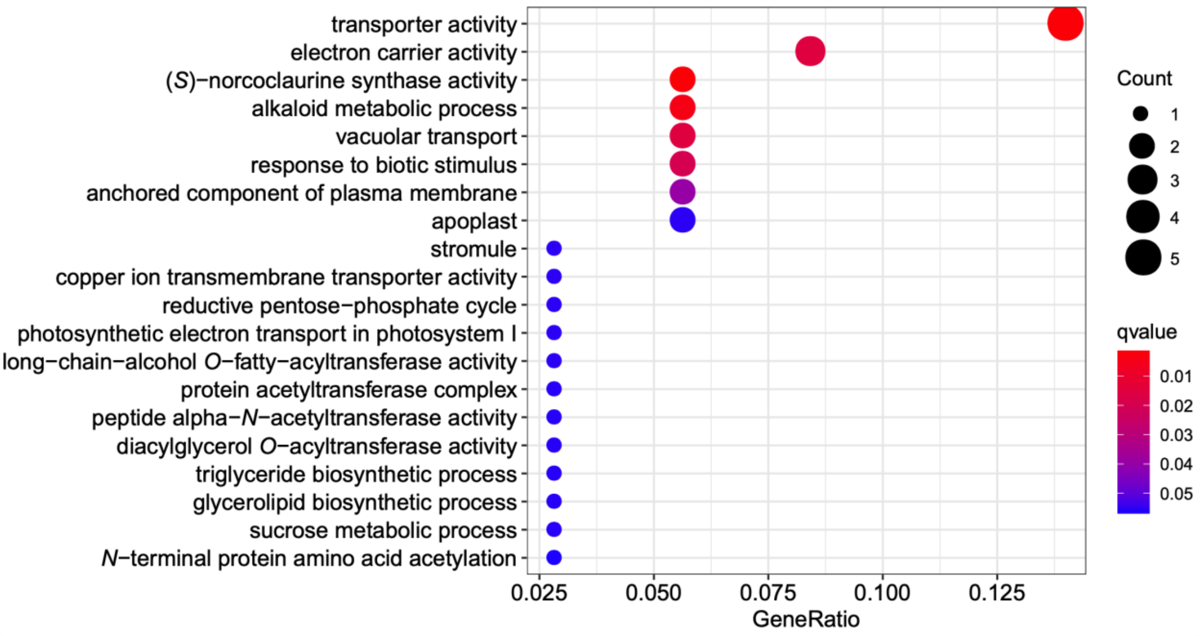

Supplementary Fig. 11. Gene Ontology enrichment analysis of genes specifically presented in the Ranunculales clade.

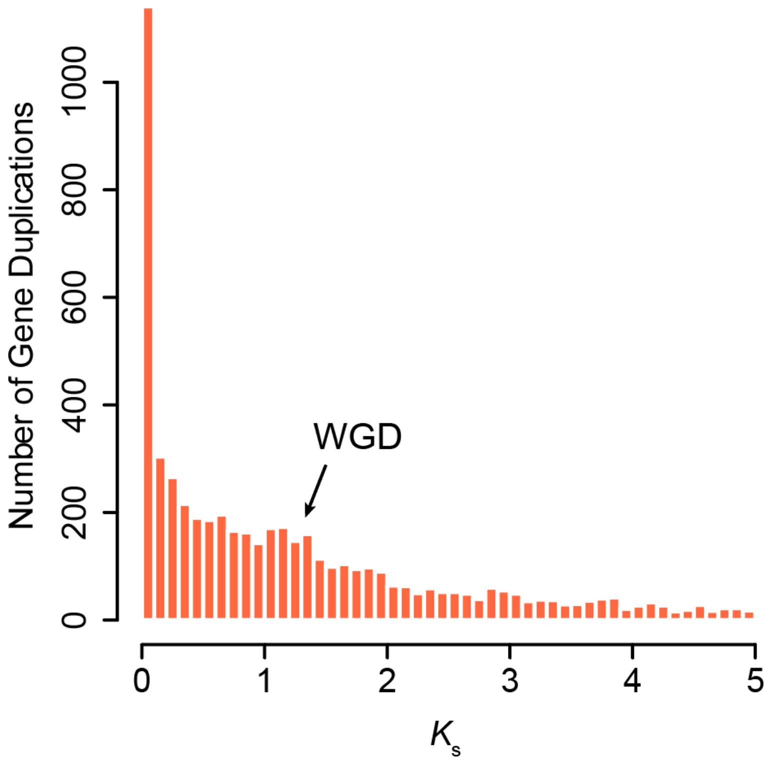

Supplementary Fig. 12. Histogram distribution of synonymous divergence (K_s) for duplicated genes of *Coptis teeta* (transcriptome data with *k*-mer= 35). A whole-genome duplication (WGD) event was indicated at about K_s = 1.08.

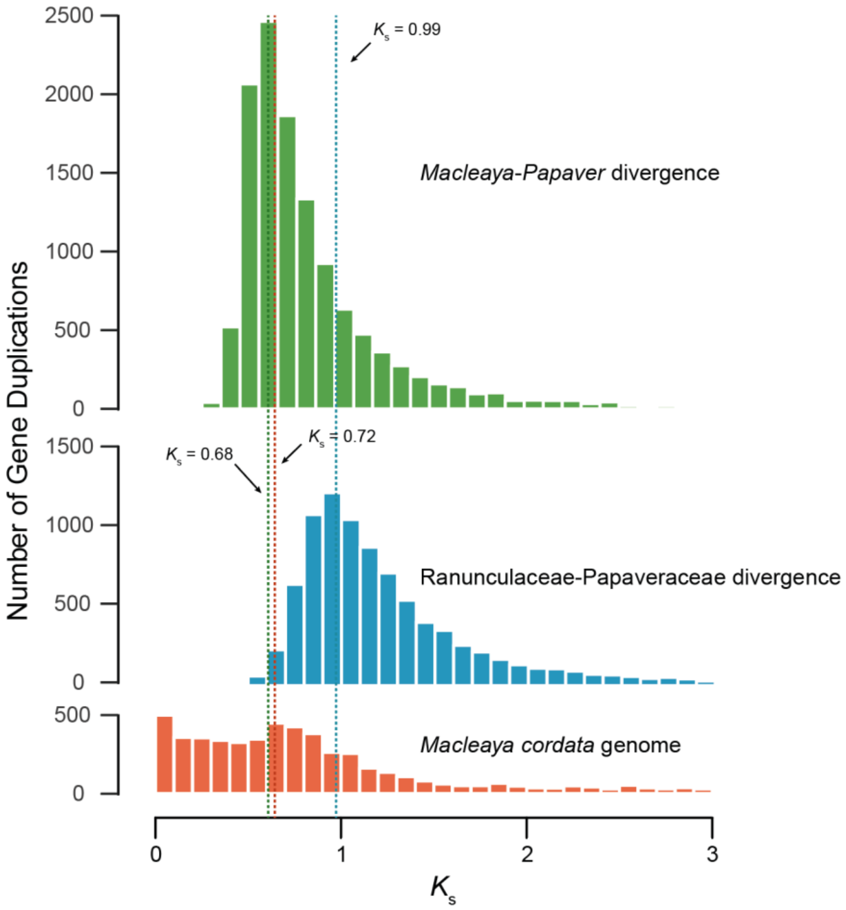

Supplementary Fig. 13. K_s plots and ortholog divergence between *Coptis chinensis*, *Macleaya cordata* and *Papaver somniferum*. A shared Papaveraceae whole-genome duplication event (K_s 0.72) is found to be older than the *Macleaya* and *Papaver* divergence (K_s 0.68), but predate the Ranunculaceae and Papaveraceae divergence (K_s 0.99).

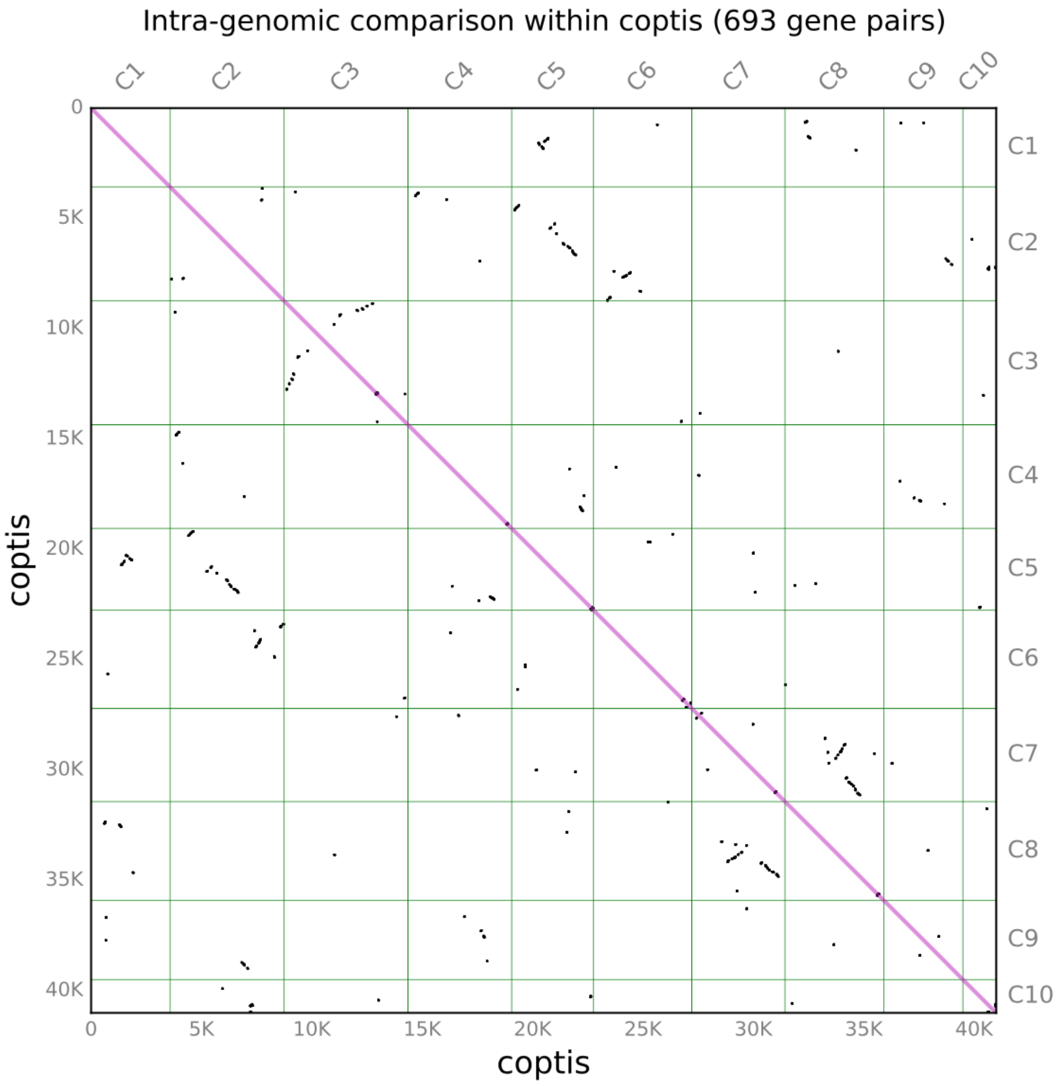

Supplementary Fig. 14. Dotplot of synteny blocks within the *Coptis chinensis* genome. C1-C9 are nine chromosome-scale pseudomolecules, C10 is the set of unanchored contigs.

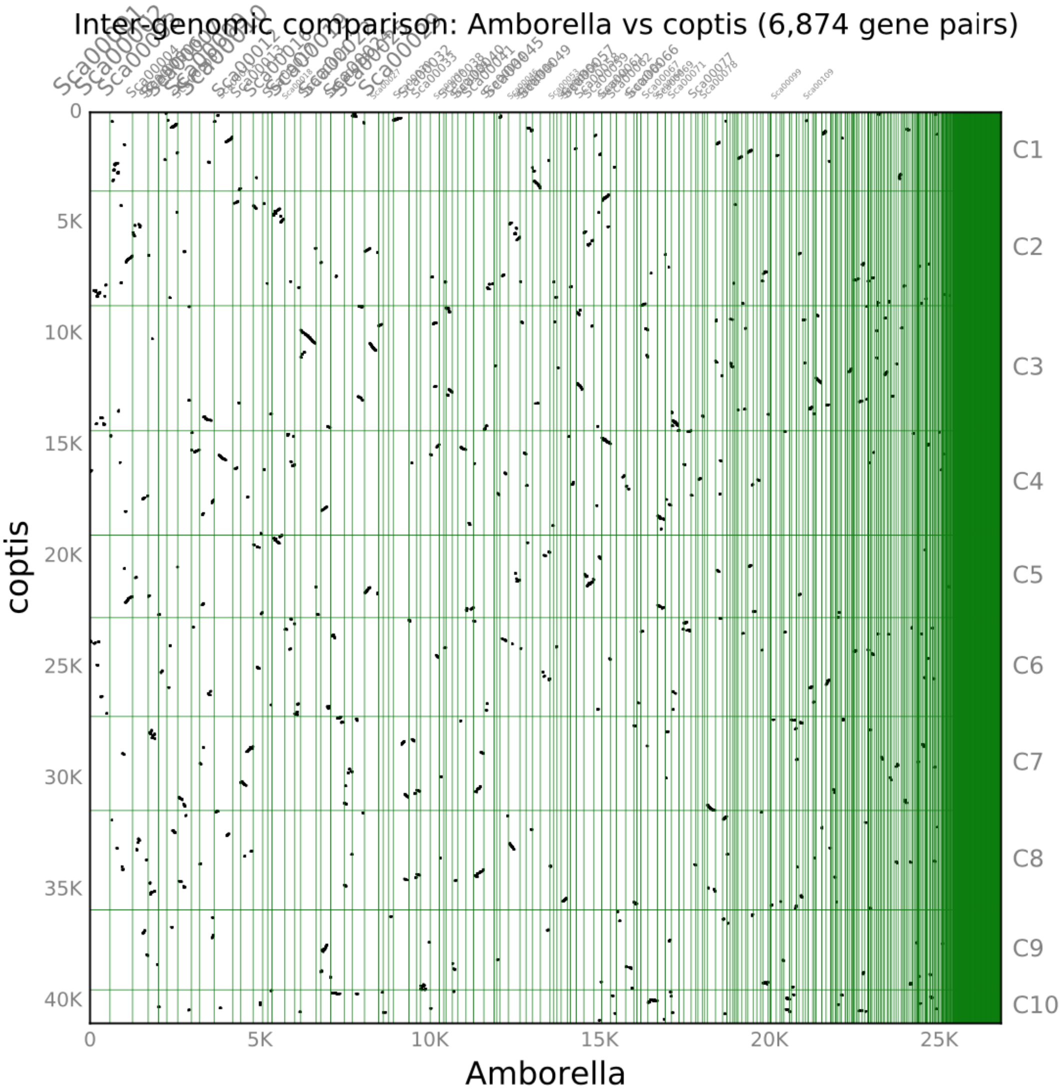

Supplementary Fig. 15. Syntenic dotplot illustrating the comparative analysis of the *Coptis chinensis* and *Amborella trichopoda g*enomes. C1-C9 are nine chromosome-scale pseudomolecules, C10 is the set of unanchored contigs of *C. chinensis* genome assembly.

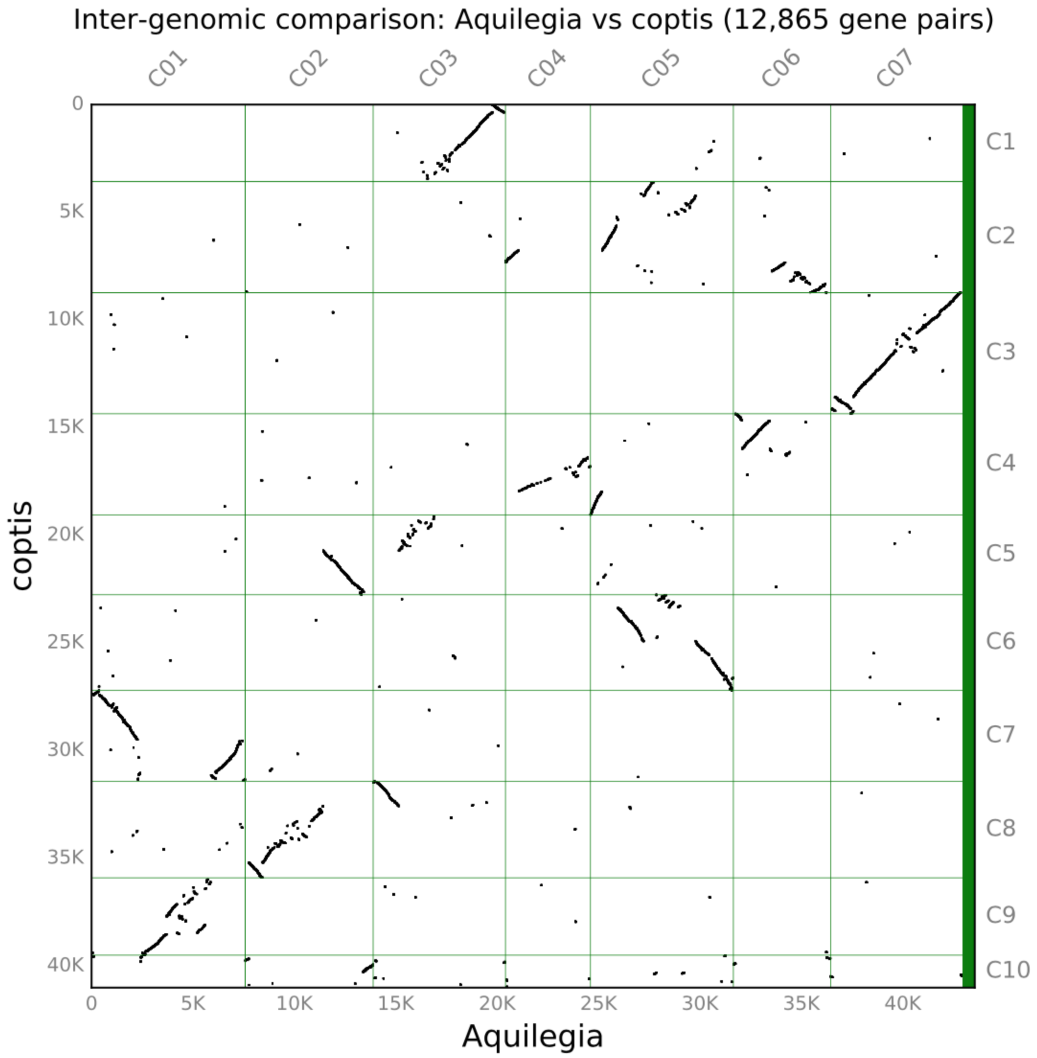

Supplementary Fig. 16. Syntenic dotplot illustrating the comparative analysis of the *Coptis chinensis* and *Aquilegia coerulea* genomes. C1-C9 are nine chromosome-scale pseudomolecules, C10 is the set of unanchored contigs of *C. chinensis* genome assembly.

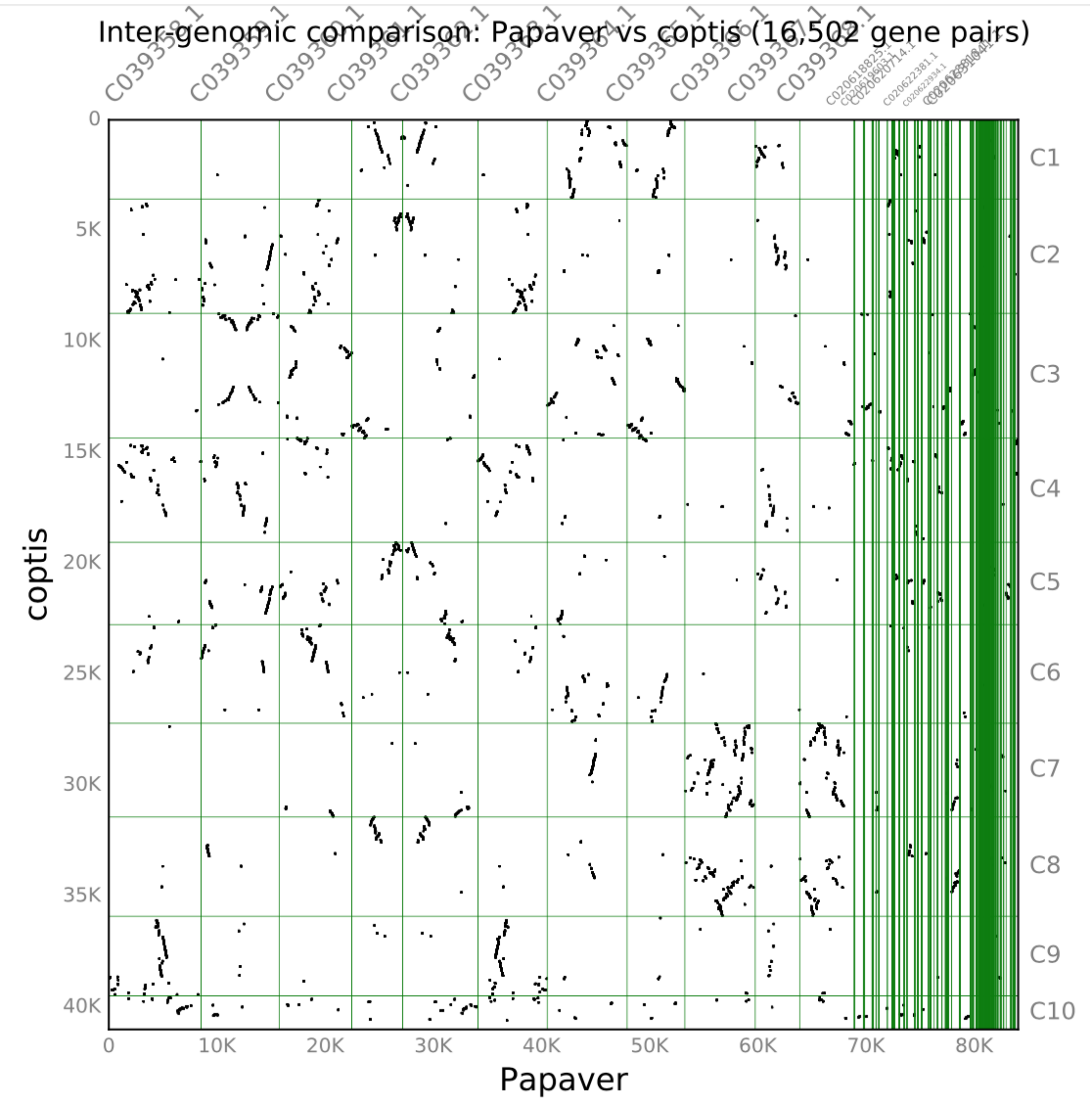

Supplementary Fig. 17. Syntenic dotplot illustrating the comparative analysis of the *Coptis chinensis* and *Papaver somniferum* genomes. C1-C9 are nine chromosome-scale pseudomolecules, C10 is the set of unanchored contigs of *C. chinensis* genome assembly.

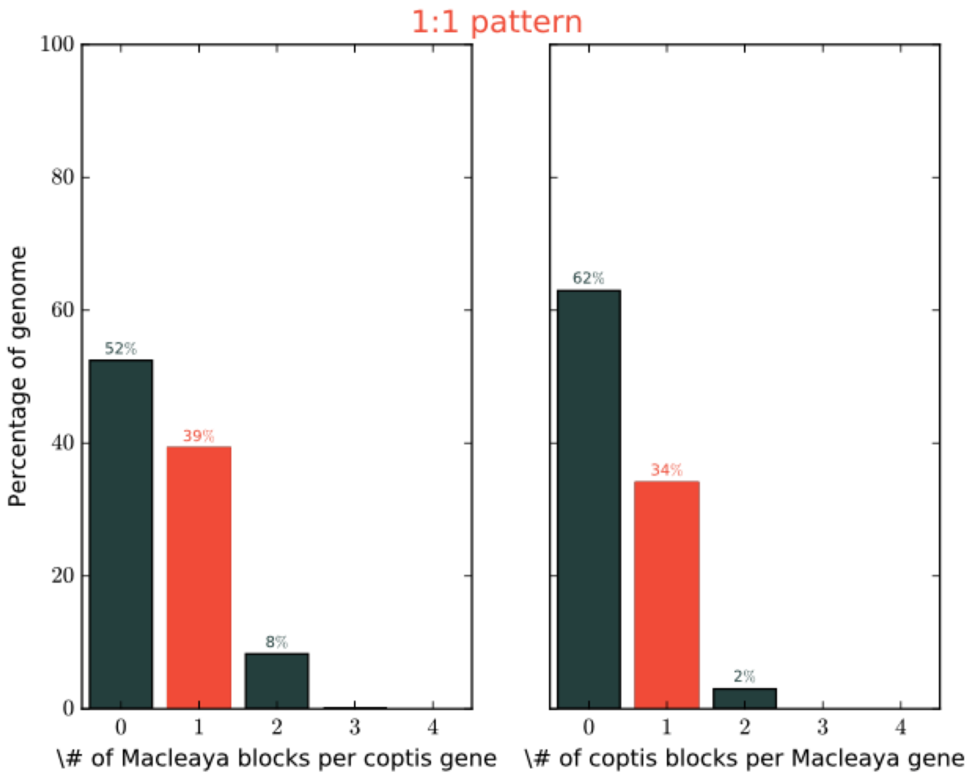

Supplementary Fig. 18. The syntenic depth ratio between the *Coptis chinensis* and *Macleaya cordata* genomes.

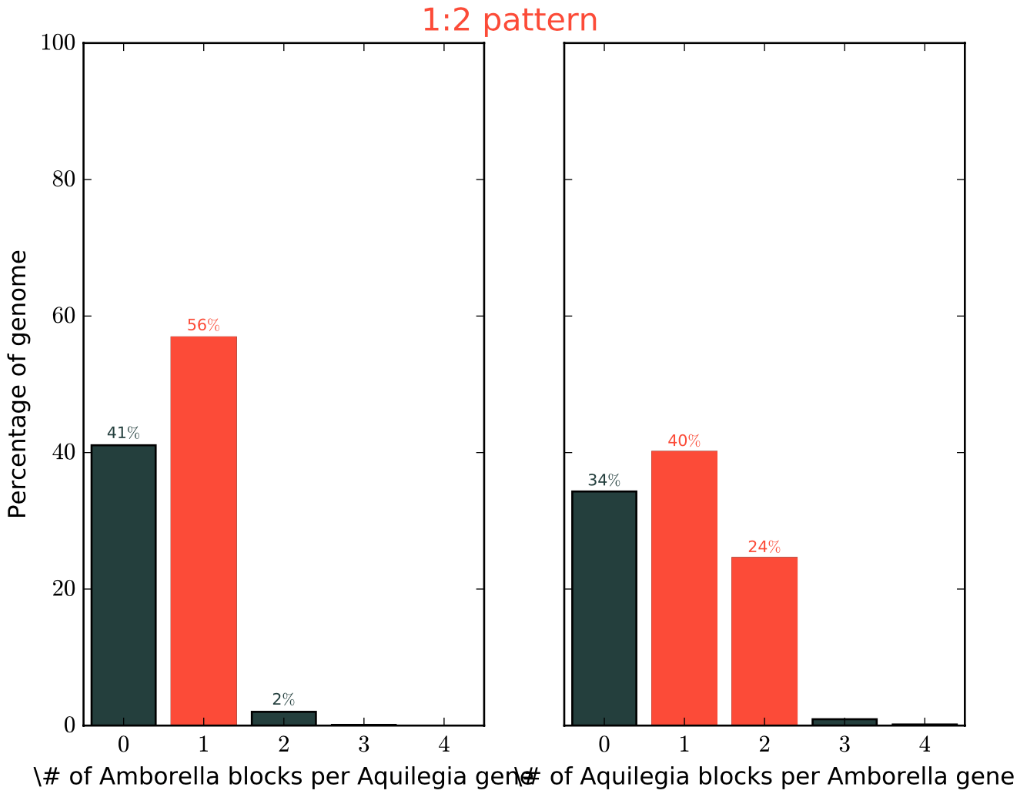

Supplementary Fig. 19. The syntenic depth ratio between the *Aquilegia coerulea* and *Amborella trichopoda* genomes.

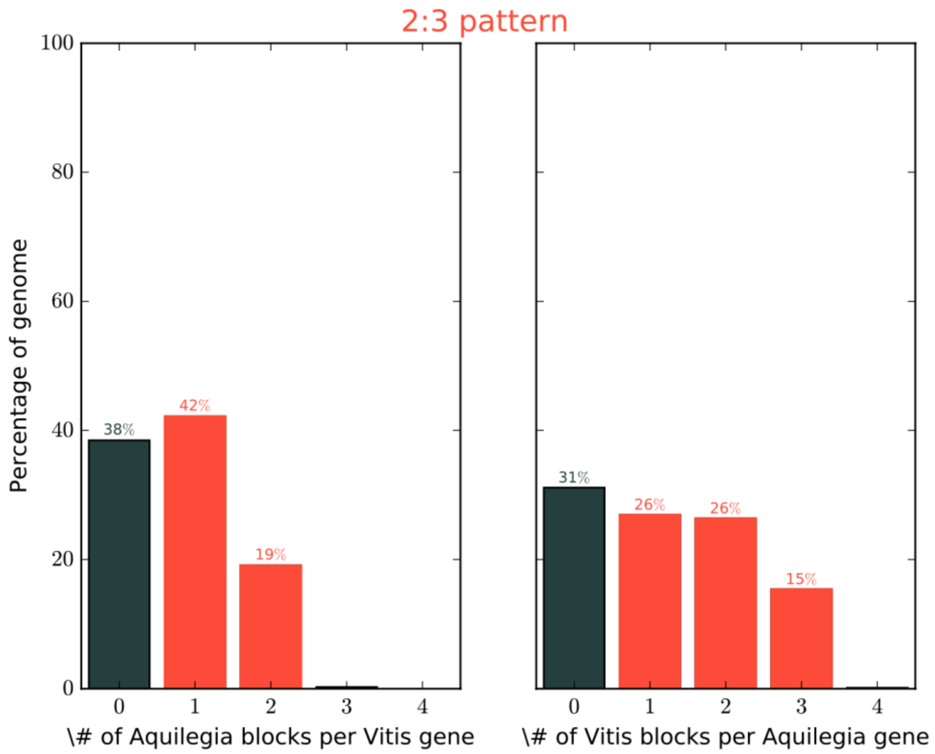

Supplementary Fig. 20. The syntenic depth ratio between the *Aquilegia coerulea* and *Vitis vinifera* genomes.

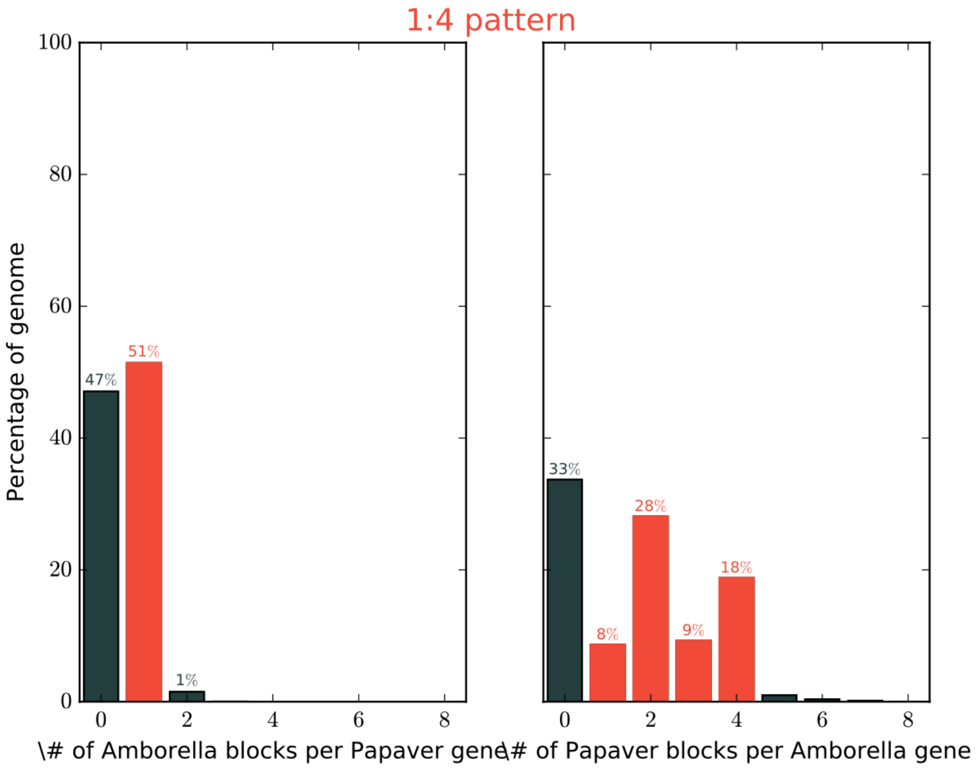

Supplementary Fig. 21. The syntenic depth ratio between the *Papaver somniferum* and *Amborella trichopoda* genomes.

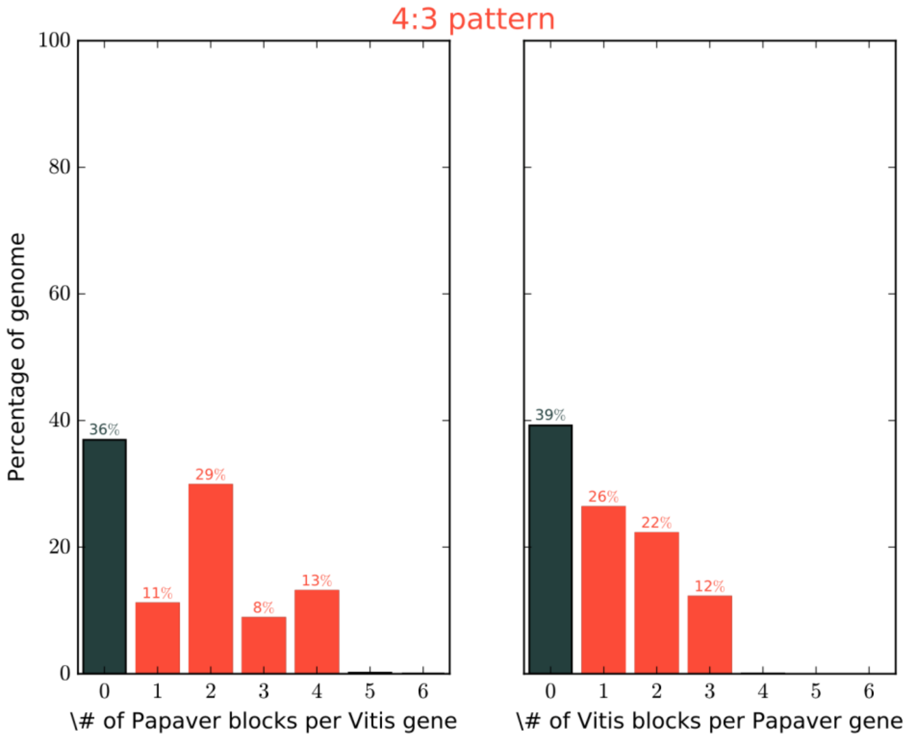

Supplementary Fig. 22. The syntenic depth ratio between the *Papaver somniferum* and *Vitis vinifera* genomes.

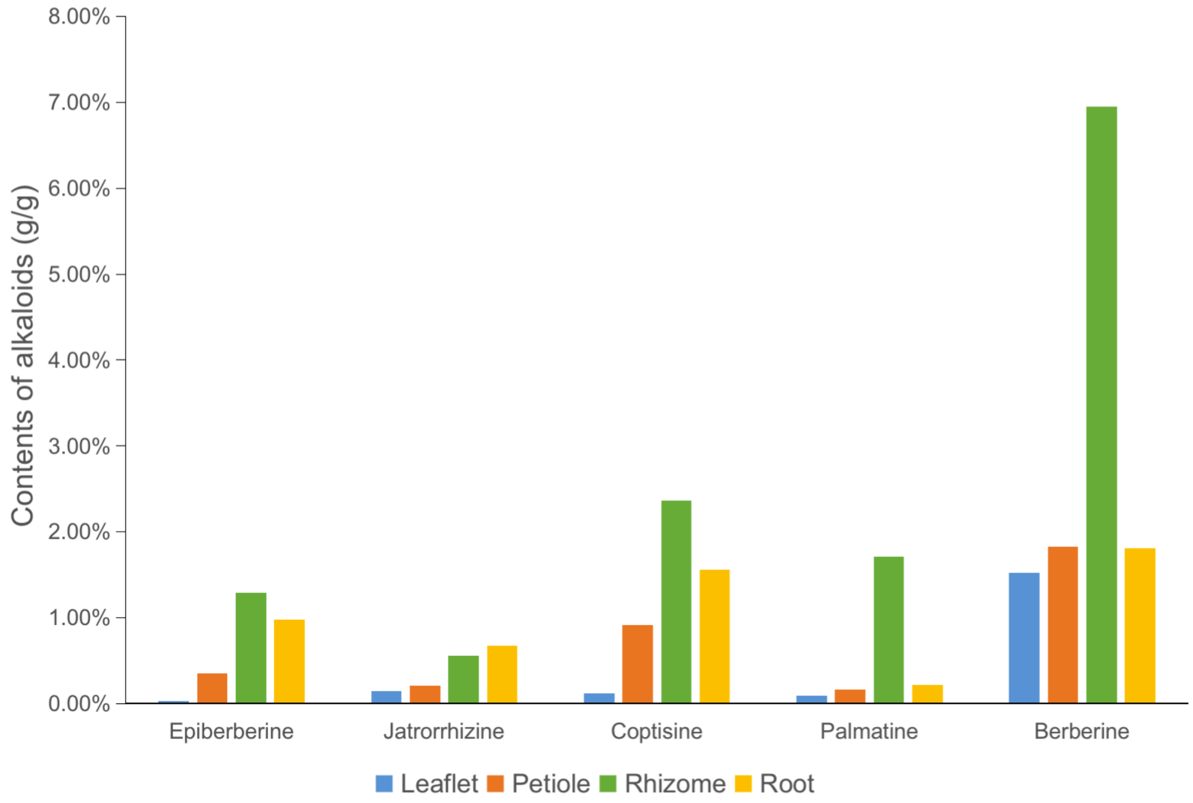

Supplementary Fig. 23. The distribution of the identified benzylisoquinoline alkaloids in four different tissues of *Coptis chinensis*. Source data are provided as a Source Data file.

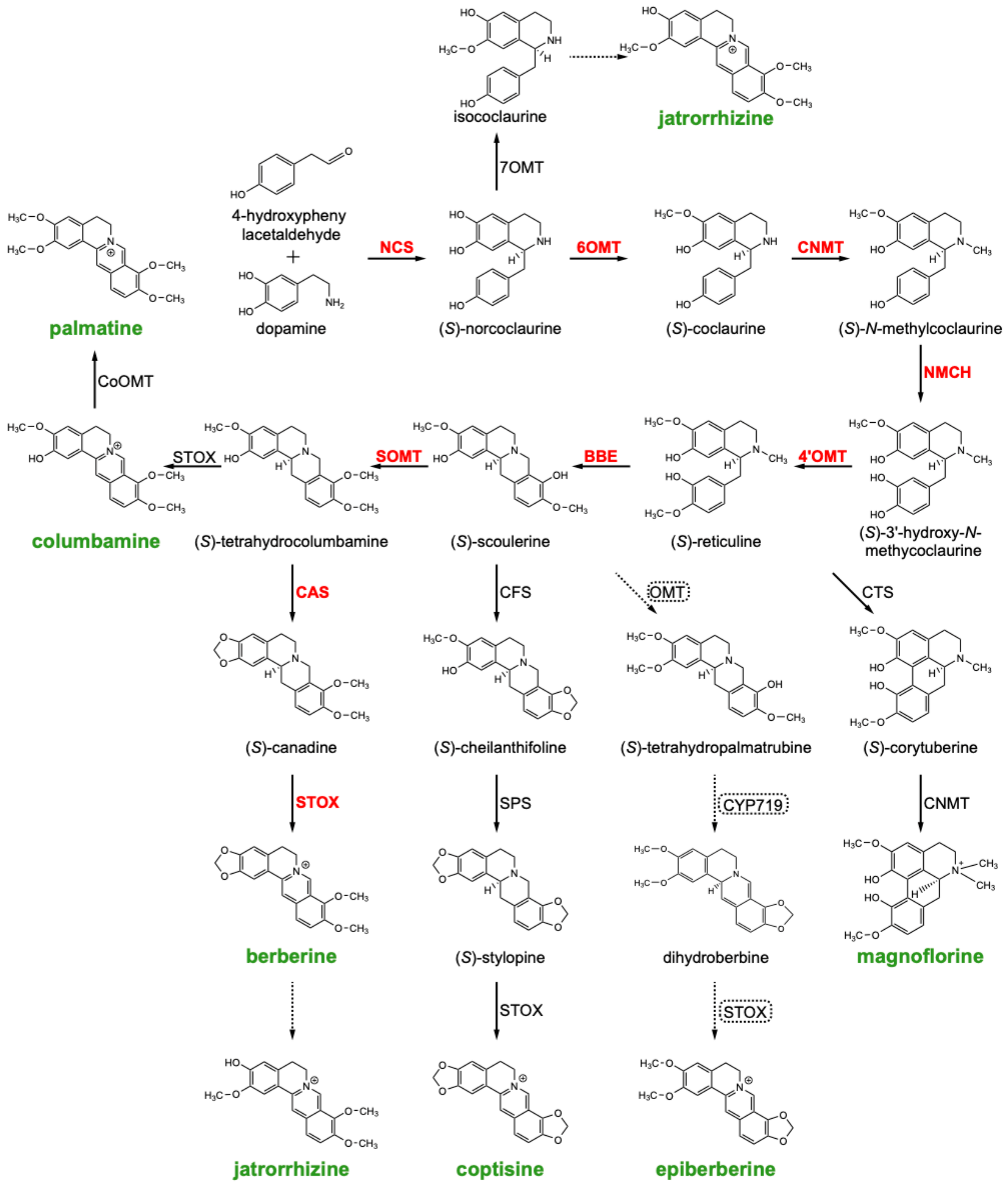

Supplementary Fig. 24. Putative biosynthetic pathways of protoberberine-type alkaloids in *Coptis*. Full names of enzymes were showed in Supplementary Data 5. The enzymes involved in the berberine pathway are marked in red and the suspected biosynthesis steps are indicated by dashed arrows.

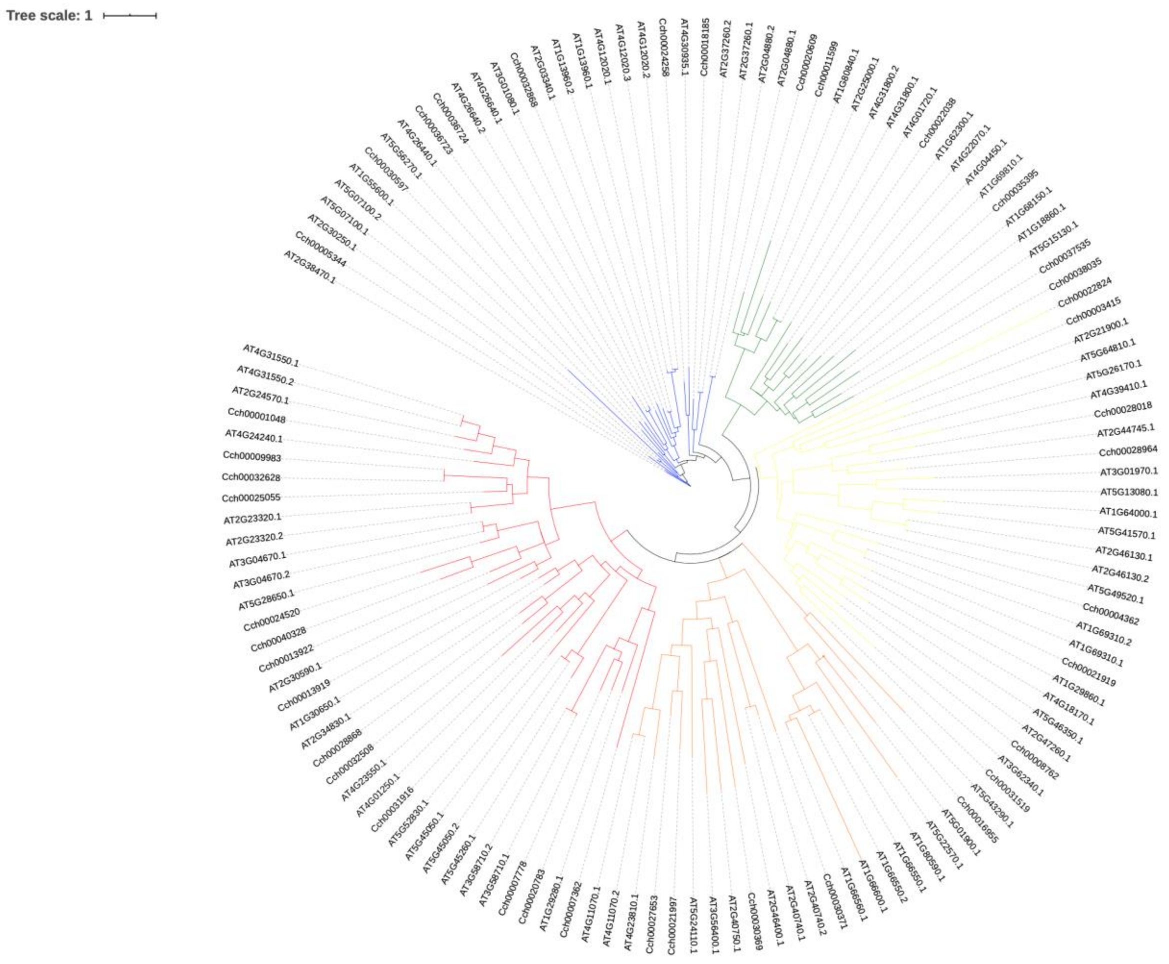

Supplementary Fig. 25. The neighbor-joining tree of all WRKY genes identified in the *Coptis chinensis* genome.

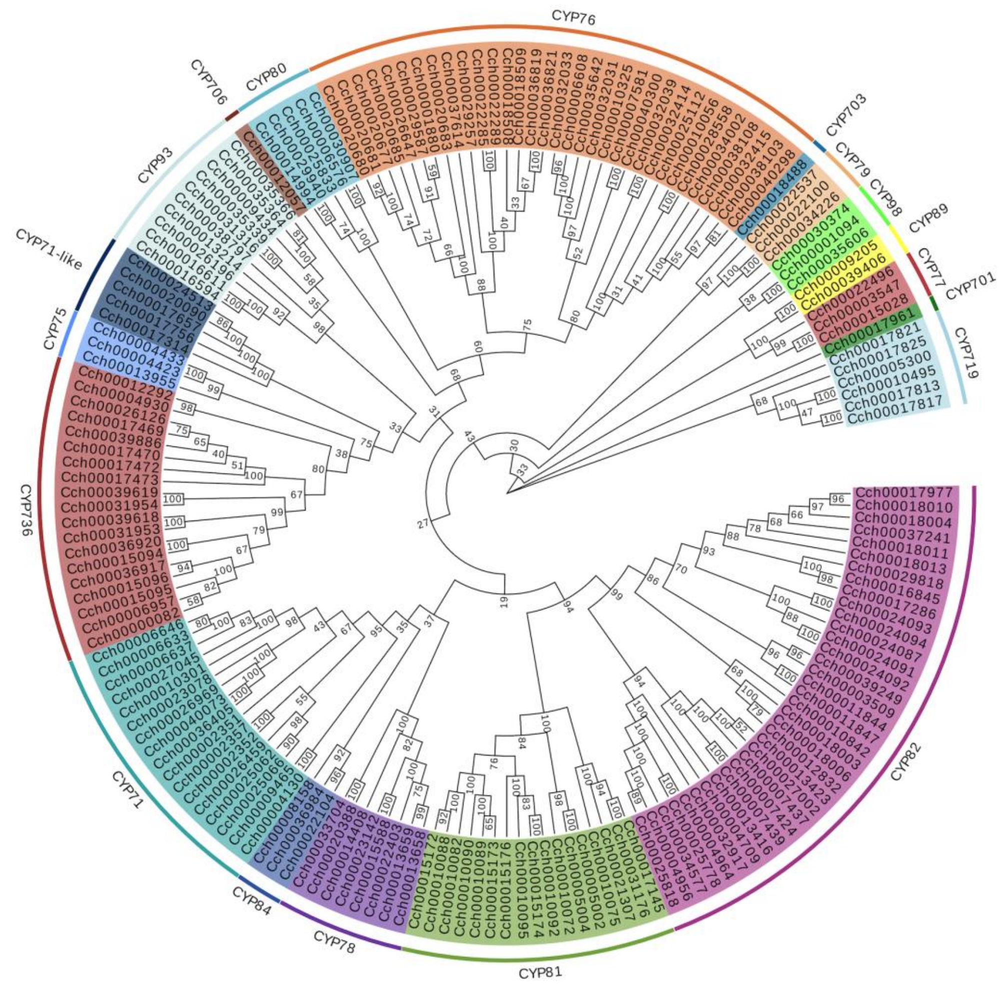

Supplementary Fig. 26. The neighbor-joining tree of all identified CYP genes belonging to the 71 clan in the *Coptis chinensis* genome.

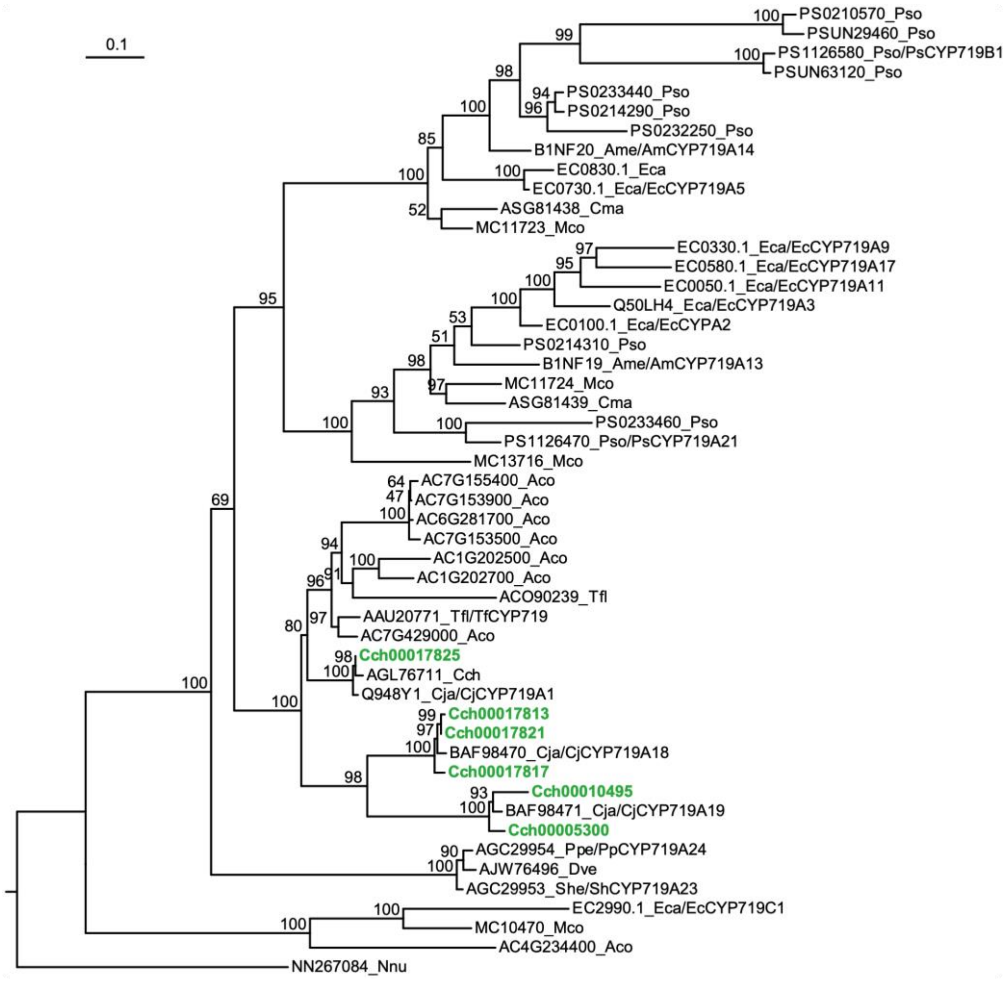

Supplementary Fig. 27. A maximum-likelihood gene tree reflecting the evolutionary relationship of CYP719 members derived from different plant species. The CYP719 genes from *Coptis chinensis* are marked in green. Other sequences are the same as those in the Figure 4 of Li et al. (2020) (Li Y., Winzer T., He Z., and Graham I.A. 2020. Over 100 Million Years of Enzyme Evolution Underpinning the Production of Morphine in the Papaveraceae Family of Flowering Plants. Plant Comm. 1, 100029). These sequences were collected from 13 plant taxa, including Cch: *C. chinensis*, Nnu: *Nelumbo nucifera*, Aco: *Aquilegia coerulea*, Ame: *Argemone mexicana*, Cma: *Chelidonium majus*, Cja: *C. japonica*, Dve: *Dysosma versipellis*, Eca: *Eschscholzia californica*, Mco: *Macleaya cordata*, Pso: *Papaver somniferum*, Ppe: *Podophyllum peltatum*, She: *Sinopodophyllum hexandrum*, Tfl: *Thalictrum flavum*.

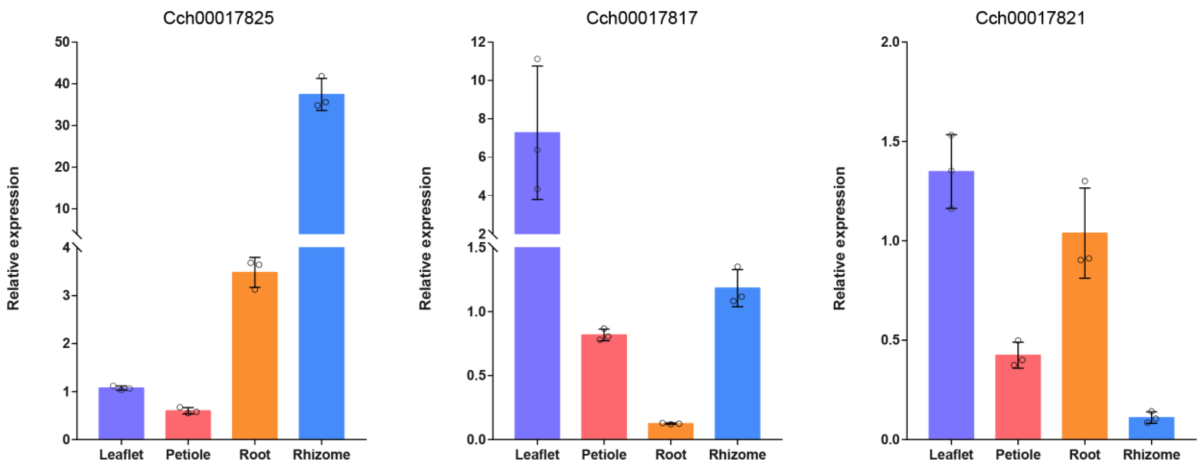

Supplementary Fig. 28. qRT-PCR validation analysis of the three expressed CYP719 genes in our transcriptome sequencing of *Coptis chinensis*. See Fig. 4b for the transcriptome derived gene expression profiles of these genes in four different tissues. Error bars, mean ± s.d.. For each gene and tissue, n = 3 independent experiments were conducted. Source data are provided as a Source Data file.

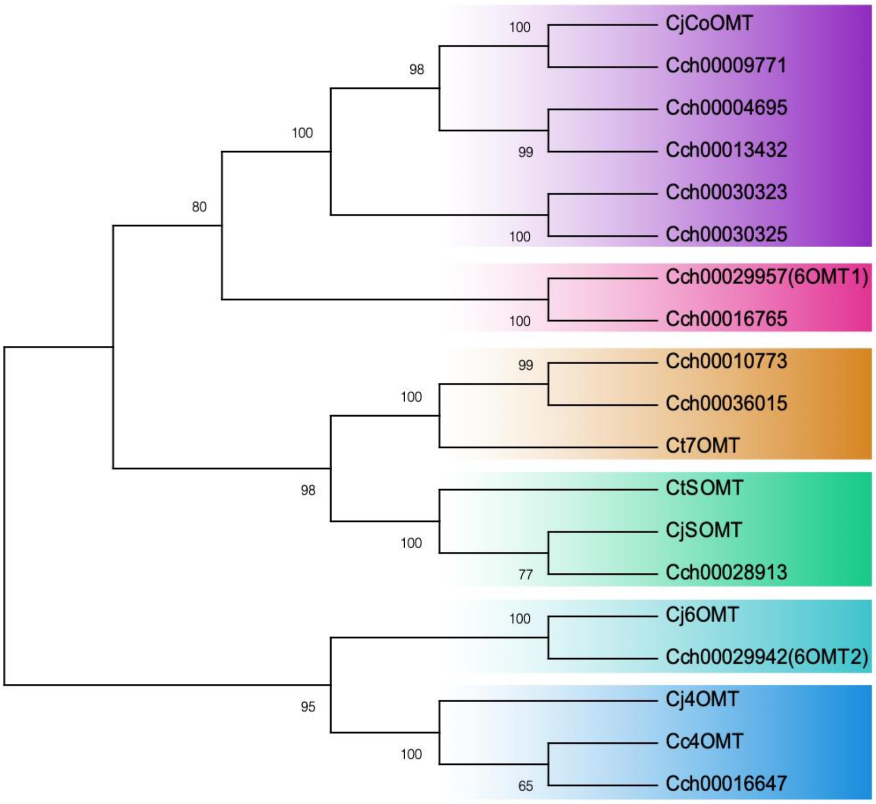

Supplementary Fig. 29. The genetic relationships of genes encoding *O*-methyltransferase in *Coptis* plants. The accession numbers for the sequences retrived from NCBI web as follows: Cj6OMT: D29811.1; Cc6OMT1:MH165875.1; Cc6OMT2: MH165876.1; Ct7OMT: MH165877.1; CjSOMT: D29809.1; CtSOMT: MH165874.1; CjCoOMT: Q8H9A8.1; Cj4'OMT1: D29812.1; Cc4'OMT: EU236699.1.

Supplementary Table 1. Summary of Illumina short reads used for assembling and polishing *Coptis chinensis* genome.

**Sample**	**Length**	**Raw reads**	**Clean reads**	**Raw base (G)**	**Clean base (G)**	**Q20(%)**	**Q30(%)**	**GC content (%)**
CC-1	150:150	144,683,821	144,463,456	43.41	43.34	96.12	89.97	38.91
CC-2	150:150	219,168,494	218,653,822	65.75	65.60	97.68	93.70	38.47

Supplementary Table 2. Statistics of the *Coptis chinensis* genome assembly.

**Assembly feature**	**Size (bp)**	**Number**
N90	254,627	1,139
N80	379,804	840
N70	519,969	628
N60	655,482	469
N50	806,550	341
Longest	4,843,910	/
Total size	936,644,440	/
Total number	/	1,801

Supplementary Table 3. Length and scaffolds for chromosome-scale pseudomolecules.

**Pseudo-chromosome**	**Size (bp)**	**Contig number**
Chr1	89,969,453	1
Chr2	114,376,183	1
Chr3	124,611,609	1
Chr4	108,390,809	1
Chr5	98,746,328	1
Chr6	100,393,100	1
Chr7	97,188,538	1
Chr8	97,686,796	1
Chr9	85,171,767	1
unmapped	20,109,857	108

Supplementary Table 4. Assembled transcripts used for validation and construction of the *Coptis chinensis* gene models.

Dataset	Number	Total length (bp)	Bases covered by assembly (%)	Sequences covered by assembly (%)	With >90% sequence in one scaffold		With >50% sequence in one scaffold	
					Number	Percentage (%)	Number	Percentage (%)
All	146661	174672120	92.3	93.5	91780	62.6	130712	89.1
>200bp	146661	174672120	92.3	93.5	91780	62.6	130712	89.1
>500bp	93595	157598341	93.5	99.1	61385	65.6	88203	94.2
>1 kb	60616	133936940	93.8	99.5	41159	67.9	57533	94.9

Supplementary Table 5. Assessing *Coptis chinensis* genome and annotation completeness with Benchmarking Universal Single-Copy Orthologs (BUSCO) analysis.

**BUSCO notation**	**Number**	**Percent**
Complete BUSCOs	277	91.5%
Complete and single-copy BUSCOs	192	63.4%
Complete and duplicated BUSCOs	85	28.1%
Fragmented BUSCOs	21	6.9%
Missing BUSCOs	5	1.6%
Total	303	100%

Supplementary Table 6. Statistics of genes annotated in the *Coptis chinensis* genome.

**Item**	**Number**
The total number of genes	41,004
The average mRNA length (bp)	3,571.75
The average coding-sequence length (bp)	968.89
The average exons per gene	4.63
The average exon length (bp)	209.38
The total number of exons	189,743
The average intron length (bp)	629.00
The total number of introns	148,739
The total intron length (bp)	93,556,632

Supplementary Table 7. The annotated genes of *Coptis chinensis* which can be functionally classified in each corresponding database.

**Database**	**Number**	**Percentage**
InterPro	32,628	79.57%
KEGG	33,836	82.52%
NR	34,075	83.10%
Uniprot	33,816	82.47%
Total	35,748	87.18%

Supplementary Table 8. Noncoding RNA genes annotated in the *Coptis chinensis* genome.

Type	Copy number	Average length (bp)	Total length (bp)
miRNA	106	115.17	12,221
tRNA	1,134	77.49	104,149
rRNA	492	300.18	147,689
snRNA	1,429	111.26	158,986

Supplementary Table 9. Statistics of repetitive element content in the *Coptis chinensis* genome.

Item	Subfamily	Number	Length (bp)	Coverage
SINE	/	260	20,271	0.00%
LINE	/	36,050	25,205,257	2.69%
LTR	/	264,701	384,363,878	41.04%
	Gypsy	214,086	332,392,923	35.49%
	Copia	47,274	50,382,058	5.38%
DNA	/	111,812	49,968,446	5.33%
Satellite	/	973	115,146	0.01%
Simple repeat	/	156,079	8,005,552	0.85%
Low complexity	/	25,374	1,218,716	0.13%
Other	/	15,036	8,835,069	0.94%
Unknown	/	319,509	114,704,336	12.25%
Total	/	929,794	585,005,427	62.46%

Supplementary Table 10. Plant genomes for phylogenetic and comparative genomics analyses.

**Plant taxon**	**Short name**	**Reference**
Arabidopsis thaliana	*Arabidopsis*	https://www.arabidopsis.org/
Theobroma cacao	cacao	https://cocoa-genome-hub.southgreen.fr/
Vitis vinifera	grape	http://www.genoscope.cns.fr/externe/GenomeBrowser/Vitis/
Aquilegia coerulea	*Aquilegia*	https://www.ncbi.nlm.nih.gov/Traces
Coptis chinensis	*Coptis*	The present study
Papaver somniferum	*Papaver*	https://www.ncbi.nlm.nih.gov/bioproject/PRJNA435796
Macleaya cordata	*Macleaya*	https://www.ncbi.nlm.nih.gov/nuccore/MVGT00000000.1/
Nelumbo nucifera	lotus	http://nelumbo.biocloud.net
Cinnamomum kanehirae	*Cinnamomum*	https://www.ncbi.nlm.nih.gov/bioproject/?term=PRJNA477266
Liriodendron chinense	*Liriodendron*	https://www.ncbi.nlm.nih.gov/bioproject/PRJNA418360
Oryza sativa	rice	http://rice.plantbiology.msu.edu/
Amborella trichopoda	*Amborella*	https://genomevolution.org/CoGe/SearchResults.pl?s=amborella&p=genome

Supplementary Table 11. Comparisons of genes and gene families among plant species investigated.

**Species**	**Genes number**	**Genes in families**	**Unclustered genes**	**Family number**	**Unique families**	**Average genes per family**
Arabidopsis	27,562	22,863	4,699	14,044	954	1.63
Cacao	21,518	19,592	1,926	14,814	310	1.32
Grape	25,834	23,104	2,730	15,038	480	1.54
Aquilegia	24,823	20,232	4,591	14,619	622	1.38
Coptis	41,004	28,926	12,078	15,984	2,397	1.81
Papaver	63,018	57,315	5,703	17,043	2,911	3.36
Macleaya	21,911	18,740	3,171	14,278	319	1.31
Lotus	46,712	31,474	15,238	15,610	1,595	2.02
Cinnamomum	26,531	21,411	5,120	13,440	631	1.59
Liriodendron	35,269	30,040	5,229	13,606	891	2.21
Rice	39,049	26,341	12,708	14,189	2,329	1.86
Amborella	17,106	15,298	1,808	12,898	269	1.19

Supplementary Table 12. The identified peaks by the mixture model on the gene age distribution (K_s) of different species genomic data.

#	Species	Family	Median K_s 1	WGD 1	Median K_s 2	WGD 2
1	*Aquilegia coerulea*	Ranunculaceae	0.9026	AQCOα		
2	*Coptis chinensis* genome	Ranunculaceae	1.083	AQCOα		
3	*Coptis chinensis* transcriptome	Ranunculaceae	1.043	AQCOα		
4	*Coptis teeta* transcriptome	Ranunculaceae	1.123	AQCOα		
5	*Papaver somniferum*	Papaveraceae	0.0834	PASOα	1.3168	PASOβ
6	*Macleaya cordata*	Papaveraceae	0.7152	PASOβ		
7	*Nelumbo nucifera*	Nelumbonaceae	0.4668	NENUα		

Supplementary Table 13. The ortholog divergence between different species genome pairs investigated.

#	Taxon 1	Taxon 2	Mean	Median	SD	Minimum K_s ortholog divergence	Maximum K_s ortholog divergence
1	*Aquilegia coerulea*	*Coptis chinensis*	0.6819	0.6708	0.1438	0.0001	1
2	*Aquilegia coerulea*	*Papaver somniferum*	1.3575	1.3225	0.2916	0.7	2
3	*Coptis chinensis*	*Nelumbo nucifera*	1.3209	1.2313	0.4017	0.5	2.5
4	*Nelumbo nucifera*	*Papaver somniferum*	1.4257	1.3422	0.4015	0.5	2.5
5	*Papaver somniferum*	*Coptis chinensis*	1.4587	1.3848	0.4089	0.5	2.5
6	*Macleaya cordata*	*Papaver somniferum*	0.7220	0.6761	0.2098	0.1	1.3
7	*Macleaya cordata*	*Coptis chinensis*	1.0123	0.9882	0.2213	0.5	1.5
8	*Macleaya cordata*	*Aquilegia coerulea*	1.0175	0.9959	0.2178	0.0001	1.5

Supplementary Table 14. The primers used for qRT-PCR validation analysis.

Gene ID	Primer name	Primer sequence (5’ to 3’)
Cch00017825	825-F	TGGTGAGGCCACTTCTCTCT
	825-R	TCTTGTGCTCCTTGTTCACG
Cch00017821	821-F	TGGATTTGTTATTGATTGATGCT
	821-R	AATAAGCCATAGAAAACTCCCCT
Cch00017817	817-F	AGAGTTGGAGAGGTCCCGTTA
	817-R	TAATAATTAGTTGTTTAGCTT
Ccβ-Actin	Ccβ-Actin-F	GTCACACCGTCCCCATTTA
	Ccβ-Actin-R	GTCACGGACGATTTCTCGTT
