# Supplementary material for: Analysis of the Coptis chinensis genome reveals the diversification of protoberberine-type alkaloids
Source: Nat Commun. 2021 Jun 2;12:3276. doi: 10.1038/s41467-021-23611-0 (PMC8172641; doi:10.1038/s41467-021-23611-0)
Supplement: Supplementary file 3 — Description of Additional Supplementary Files [file 41467_2021_23611_MOESM3_ESM.pdf]

Description of additional supplementary files

Title: Supplementary Data 1

Description: Transposons identified which have genomic distribution close or within benzylisoquinoline alkaloid (BIA) functional genes.

Title: Supplementary Data 2

Description: Enriched KEGG pathways for gene families expanded in *Coptis chinensis*. The *p* values were computed based on two-sided hypergeometric test and corrected with Benjamini-Hochberg. The *q* values were calculated using the Benjamini-Hochberg procedure to account for multiple testing.

Title: Supplementary Data 3

Description: The null simulations of the background gene birth and death rates in each Multi-tAxon Paleopolyploidy Search (MAPS) analysis. The *p*-value for a one-sided Fisher's exact test was used to detect nodes with a significantly higher proportion of inferred gene duplications compared to the null distribution. An * indicates a significant node. No adjustment made for multiple comparison.

Title: Supplementary Data 4

Description: The positive simulations of the background gene birth and death rates in each Multi-tAxon Paleopolyploidy Search (MAPS) analysis. The *p*-value for a one-sided Fisher's exact test was used to detect a significant node which was indicated by *. No adjustment made for multiple comparison.

Title: Supplementary Data 5

Description: Location of the already-known benzylisoquinoline alkaloid metabolism genes in the *Coptis chinensis* genome.

Title: Supplementary Data 6

Description: bHLH members identified in the *Coptis chinensis* genome.

Title: Supplementary Data 7

Description: AP2/ERF members identified in the *Coptis chinensis* genome.

Title: Supplementary Data 8

Description: CYP gene families identified in *Coptis chinensis* and comparative analysis with other plants.
