# Supplementary material for: Analysis of the Coptis chinensis genome reveals the diversification of protoberberine-type alkaloids
Source: Nat Commun. 2021 Jun 2;12:3276. doi: 10.1038/s41467-021-23611-0 (PMC8172641; doi:10.1038/s41467-021-23611-0)
Supplement: Supplementary file 4 — Reporting Summary [file 41467_2021_23611_MOESM4_ESM.pdf]

nature research

Corresponding author(s): Yifei Liu, Shilin Chen, Xuekui Wang, Jing Nie

Last updated by author(s): Apr 28, 2021

Reporting Summary

Nature Research wishes to improve the reproducibility of the work that we publish. This form provides structure for consistency and transparency in reporting. For further information on Nature Research policies, see our Editorial Policies and the Editorial Policy Checklist.

Statistics

For all statistical analyses, confirm that the following items are present in the figure legend, table legend, main text, or Methods section.

n/a	Confirmed	
☐	☒	The exact sample size (*n*) for each experimental group/condition, given as a discrete number and unit of measurement
☐	☒	A statement on whether measurements were taken from distinct samples or whether the same sample was measured repeatedly
☐	☒	The statistical test(s) used AND whether they are one- or two-sided<br>*Only common tests should be described solely by name; describe more complex techniques in the Methods section.*
☒	☐	A description of all covariates tested
☐	☒	A description of any assumptions or corrections, such as tests of normality and adjustment for multiple comparisons
☐	☒	A full description of the statistical parameters including central tendency (e.g. means) or other basic estimates (e.g. regression coefficient) AND variation (e.g. standard deviation) or associated estimates of uncertainty (e.g. confidence intervals)
☐	☒	For null hypothesis testing, the test statistic (e.g. *F*, *t*, *r*) with confidence intervals, effect sizes, degrees of freedom and *P* value noted<br>*Give P values as exact values whenever suitable.*
☒	☐	For Bayesian analysis, information on the choice of priors and Markov chain Monte Carlo settings
☒	☐	For hierarchical and complex designs, identification of the appropriate level for tests and full reporting of outcomes
☐	☒	Estimates of effect sizes (e.g. Cohen's *d*, Pearson's *r*), indicating how they were calculated

Our web collection on statistics for biologists contains articles on many of the points above.

Software and code

Policy information about availability of computer code

Data collection	Oxford Nanopore Albacore software (v2.1.3), Infernal (v1.1.1), Waters MassLynx software (v4.1)
Data analysis	Jellyfish (v2.1.4), GenomeScope (v1.0), Canu (v1.6), SMARTdenovo (v1.5), Pilon (v1.22), BWA (v0.7.17), BUSCO (v2.0), Trinity (v2.3.3), Maker package (v2.31.10), Augustus (v3.2.1), RNAmmer (v1.2), tRNAscan-SE (v1.23), Rfam_scan.pl (v1.0.4), BLAST (v0.7.9), InterProScan (v5.33), KOBAS (v2.0), RepeatModeler (v1.0.4), RepeatMasker (v4.0.5), PicardTools (v1.95), SAMtools (v1.11), BCFtools (v1.7), PSMC, MUSCLE (v3.8.31), RAxML (v8.2.10), ASTRAL-III, MCMCtree(v4.0), PAML(v4.8), OrthoMCL (v2.0.5), CAFÉ (v4.1), MCScan (Python-version), LAST, MAPS, PASTA, plantiSMASH, SMART, MEGAX (10.1.8), R (v3.6.3), Python (v3.7), Perl (5.26.2). Specific parameters used during run-time are provided in the methods. All softwares or scripts are available from official websites or GitHub as indicated in the methods.

For manuscripts utilizing custom algorithms or software that are central to the research but not yet described in published literature, software must be made available to editors and reviewers. We strongly encourage code deposition in a community repository (e.g. GitHub). See the Nature Research guidelines for submitting code & software for further information.

Data

Policy information about availability of data

All manuscripts must include a data availability statement. This statement should provide the following information, where applicable:
- Accession codes, unique identifiers, or web links for publicly available datasets
- A list of figures that have associated raw data
- A description of any restrictions on data availability

The raw sequence reads and genome assembly have been deposited in NCBI under the BioProject accession number PRJNA662860 and BioSample accessions SAMN18434929-SAMN18434940

Field-specific reporting

Please select the one below that is the best fit for your research. If you are not sure, read the appropriate sections before making your selection.

☒ Life sciences ☐ Behavioural & social sciences ☐ Ecological, evolutionary & environmental sciences

For a reference copy of the document with all sections, see nature.com/documents/nr-reporting-summary-flat.pdf

Life sciences study design

All studies must disclose on these points even when the disclosure is negative.

Sample size	One sample of Coptis chinensis was used for genome sequencing. For RNA-seq three biological samples were used. For experiments determing metabolic contents three biological independent samples were used. All these samples were wild-type and were collected from the planting areas of Coptis.
Data exclusions	For genome assembly, sequences with low-quality were excluded.
Replication	For RNA-seq, three biological replicates were used, and for metabolic analysis, three biological replicates were used.
Randomization	No random sampling is required for genome sequencing, because the genome differences are very small within the wild population, thus any wild plant is allowed for genome sequencing.
Blinding	Blinding is not applicable in our study because it does not involve subjects which receive different treatments. All experiments were done by analyzing data derived from different biological replicates directly.

Reporting for specific materials, systems and methods

We require information from authors about some types of materials, experimental systems and methods used in many studies. Here, indicate whether each material, system or method listed is relevant to your study. If you are not sure if a list item applies to your research, read the appropriate section before selecting a response.

Materials & experimental systems

n/a	Involved in the study	
☒	☐	Antibodies
☒	☐	Eukaryotic cell lines
☒	☐	Palaeontology and archaeology
☒	☐	Animals and other organisms
☒	☐	Human research participants
☒	☐	Clinical data
☒	☐	Dual use research of concern

Methods

n/a	Involved in the study	
☒	☐	ChIP-seq
☐	☒	Flow cytometry
☒	☐	MRI-based neuroimaging

Flow Cytometry

Plots

Confirm that:

☒ The axis labels state the marker and fluorochrome used (e.g. CD4-FITC).

☒ The axis scales are clearly visible. Include numbers along axes only for bottom left plot of group (a 'group' is an analysis of identical markers).

☒ All plots are contour plots with outliers or pseudocolor plots.

☒ A numerical value for number of cells or percentage (with statistics) is provided.

Methodology

Sample preparation	Nuclei were isolated from young leaves and stained by PI for 15 minutes
Instrument	BD Accuri C6
Software	BD Accuri C6

Cell population abundance	Abundance >10000 cells were collected for a sample. Total nuclei populations were gated using relative fluorescence intensity.
Gating strategy	Total nuclei populations were gated using PI intensity. In PI+ singles cells, the proportions of nuclei of a sample and its contrast were determined based on their PI intensity

☒ Tick this box to confirm that a figure exemplifying the gating strategy is provided in the Supplementary Information.
